# Reward-specific satiety affects subjective value signals in orbitofrontal cortex during multicomponent economic choice

**DOI:** 10.1073/pnas.2022650118

**Published:** 2021-07-20

**Authors:** Alexandre Pastor-Bernier, Arkadiusz Stasiak, Wolfram Schultz

**Affiliations:** ^a^Department of Physiology, Development and Neuroscience, University of Cambridge, Cambridge CB2 3DY, United Kingdom

**Keywords:** bundle, multicomponent choice, stochastic choice, choice indifference, revealed preference

## Abstract

Sensitivity to satiety constitutes a basic requirement for neuronal coding of subjective reward value. Satiety from natural ongoing consumption affects reward functions in learning and approach behavior. More specifically, satiety reduces the subjective economic value of individual rewards during choice between options that typically contain multiple reward components. The unconfounded assessment of economic reward value requires tests at choice indifference between two options, which is difficult to achieve with sated rewards. By conceptualizing choices between options with multiple reward components (“bundles”), Revealed Preference Theory may offer a solution. Despite satiety, choices against an unaltered reference bundle may remain indifferent when the reduced value of a sated bundle reward is compensated by larger amounts of an unsated reward of the same bundle, and then the value loss of the sated reward is indicated by the amount of the added unsated reward. Here, we show psychophysically titrated choice indifference in monkeys between bundles of differently sated rewards. Neuronal chosen value signals in the orbitofrontal cortex (OFC) followed closely the subjective value change within recording periods of individual neurons. A neuronal classifier distinguishing the bundles and predicting choice substantiated the subjective value change. The choice between conventional single rewards confirmed the neuronal changes seen with two-reward bundles. Thus, reward-specific satiety reduces subjective reward value signals in OFC. With satiety being an important factor of subjective reward value, these results extend the notion of subjective economic reward value coding in OFC neurons.

## Significance

Ongoing consumption reduces the subjective value of rewards to different degrees depending on their individual properties, a phenomenon referred to as sensory-specific satiety. Such value change should be manifested in economic choices, and neuronal signals for subjective economic reward value should be sensitive to reward-specific satiety. We tested monkeys during the choice between two options that each contained two different rewards (“bundles”); the two rewards were prone to different degrees of satiety. Ongoing reward consumption affected choices in a way that indicated satiety-induced reward-specific change of subjective economic value. Neuronal responses in the monkey orbitofrontal cortex (OFC) followed the differential reduction of subjective economic value. These results satisfy a crucial requirement for subjective reward value coding in OFC neurons.

There are no specific sensory receptors for rewards, and their value is determined by the needs of individual decision makers. Thus, rewards have subjective value rather than being solely characterized by physical measures such as molecular concentrations, milliliters of juice, or units of money. Accordingly, neuronal signals in prime reward structures, such as orbitofrontal cortex (OFC) and dopamine neurons, code reward value on a subjective basis (1–3). One of the key factors determining subjective value is satiety that arises from ongoing reward consumption. After drinking a cup of coffee, we may desire a glass of water. We feel sated on coffee while still seeking liquid. Apparently, the coffee has lost more value for us than water. Such value loss is often referred to as sensory-specific satiety (or, more appropriately here, reward-specific satiety) and contrasts with general satiety that refers indiscriminately to all rewards (4, 5). Thus, reward-specific satiety is a key factor of subjective reward value, and any claim toward neuronal subjective reward value coding should include sensitivity to reward-specific satiety.

Reward-specific satiety in humans, monkeys, and rodents affects approach behavior, goal-directed behavior, operant responding, learning, and pleasantness associated with the specific reward. Lesioning, inactivation, and neural studies demonstrate the involvement of frontal cortex, and in particular OFC, in behavioral changes induced by general and reward-specific satiety. The studies assessed alterations of associative strength, cognitive representations for learning, approach behavior, and goal-directed behavior (6–18) but did not address the appreciation and maximization of subjective economic reward value that constitute the central interest of economic decision theory (18–20) and current neuroeconomics research (1, 21–24). Economic reward value cannot be measured directly but is inferred from observable choice (19–21). Value estimations are made at choice indifference between a test option and a reference option, which renders them immune to unselective general satiety and controls for confounding slope changes of choice functions (25). While choice indifference is possible with milder value differences from reward type, reward delay, reward risk, or spontaneous fluctuations (1, 3, 26), it may fail with substantial satiety when animals categorically prefer nonsated alternatives (15–17). By contrast, choice indifference becomes feasible when an added amount of unsated reward can compensate for the value loss of the sated reward. Such tests require reward options with two reward components (“bundles”). Indeed, all choice options constitute bundles; they are either single rewards with multiple components, like the taste and fluid of a cup of coffee, or contain multiple rewards, like meat and vegetables of a meal we choose.

The rationale of our experiment rests on the tenet that candidate neuronal signals for subjective economic value need to be sensitive to reward-specific satiety. We used bundles whose multiple rewards sated differentially and allowed testing at choice indifference. Using strict concepts of Revealed Preference Theory (19, 27, 28), we had demonstrated that monkeys chose rationally between two-reward bundles by satisfying completeness, transitivity, and independence of irrelevant alternatives (24). Using these validations, we now estimated the loss of economic value from reward consumption during ongoing task performance. Using two-reward bundle options, instead of single-reward options, we tested choice indifference at specifically set, constant levels. We used multiple, equally preferred indifference points (IPs) for constructing two-dimensional graphic indifference curves (IC) on which all bundles had by definition the same subjective value. The slopes of these ICs demonstrated subjective value relationships (“currency”) between two bundle rewards. Ongoing reward consumption changed the IC slopes in characteristic ways that suggested reward-specific subjective value reduction. During full recording periods of individual OFC neurons, chosen value responses tracked the IC slope changes in a systematic way that suggested a neuronal correlate for reward-specific satiety. These data support and extend the claim of subjective economic reward value coding by OFC neurons.

## Results

### Design.

**Fig. 1.**
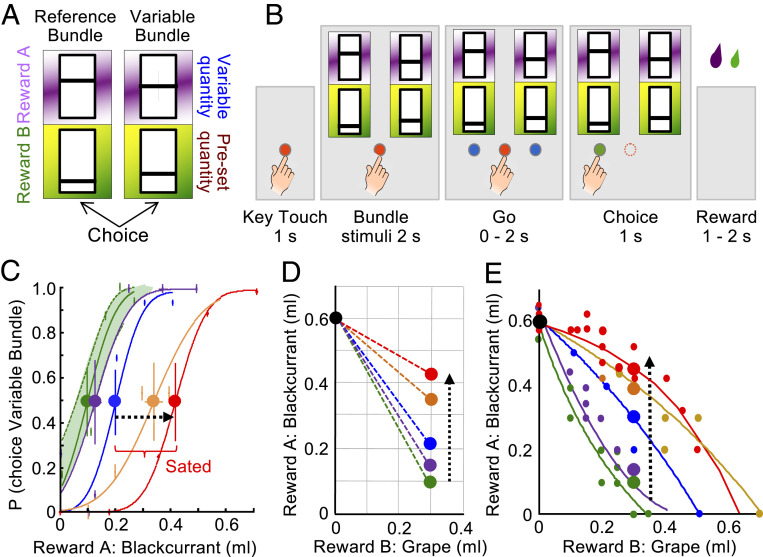
Task, design, and psychophysics. (*A*) Choice options (arbitrary reward quantity settings). Each bundle contained two rewards (Reward A and Reward B) with independently set quantities indicated by the vertical bar position within each rectangle (higher was more). The Reference Bundle contained two preset reward quantities. The Variable Bundle contained a specifically set quantity of one reward and an experimentally varied quantity of the other reward. (*B*) Task sequence: in each trial, the animal contacted a central touch key for 1.0 s, then the two choice options appeared on a computer monitor. After 2.0 s, two blue spots appeared on the monitor, and the animal touched one option within 2.0 s. After touching the target for 1.0 s, the blue spot underneath the chosen bundle turned green and the blue spot disappeared. The computer-controlled liquid solenoid valve delivered Reward A at 1.0 s after the choice and Reward B 0.5 s later. (*C*) Psychophysical assessment of choice between constant Reference Bundle (0.6 mL blackcurrant juice, 0.0 mL grape juice) and one of the Variable Bundles (varying Reward A, blackcurrant juice, from 0 to 0.7 mL, while holding grape juice constant at 0.3 mL); same bundles as *C*. Green and violet curves inside green 95% confidence interval (CI): initial choices; blue, orange, and red curves: advancing consumption; heavy dots: IP. Satiety was defined by IPs exceeding the CI. Each curve and IP were estimated from 80 trials in a single block (Weibull fits, Monkey A). (*D*) Two-dimensional map indicating progressively more blackcurrant juice required for choice indifference between the Reference Bundle and one of the Variable Bundles (from green to red); same test as *C*. Black and colored dots show bundle positions, connecting dotted lines indicate choice indifference between the connected bundles (IPs). The flattened slope indicated subjective value loss of grape juice relative to blackcurrant juice. (*E*) Gradual changes in slope and curvature of ICs between presatiety (green, violet) and during increasing satiety (blue, orange, red). Each IC was estimated from fitting to about 35 IPs (Eq. **1**), with 80 trials/IP (Monkey A). Small dots indicate IPs, and large dots indicate IPs estimated from a single psychophysical test sequence (*C* and *D*).

This study assumed that subjective reward value can be inferred from observable economic choice, that altered choice indicates changed subjective value, and that reduction of subjective value with reward consumption reflects satiety. By definition, two options that are chosen with equal probability have the same subjective value (indifference at choice *P* = 0.5 each option). Testing at choice indifference is important, as testing at other positions on the sigmoid choice function cannot exclude confounds from slope changes of the choice function (25). We implemented the following design:

1) A standard economic method estimates subjective economic value at choice indifference against a constant reference reward. As animals may not choose a sated single reward when a nonsated alternative is available (15–17) and thus fail to achieve choice indifference, we studied bundles that each contained the same two rewards on which the animals sated to different degrees. Thus, satiety-induced stronger value reduction of one reward could be compensated by larger quantity of the other, less sated reward to maintain choice indifference. Although the animal sated on one reward much less than on its alternative, it chose each bundle on one-half of the trials. The choice involved a single arm movement, and the rewards from the chosen bundle were paid out immediately. Thus, the task contained two discrete, mutually exclusive and collectively exhaustive options. Due to the statistical requirements of neuronal data analysis, we tested stochastic choice in repeated trials rather than single-shot choice.
1a) Specifically, the animal chose between a Reference Bundle and a simultaneously presented Variable Bundle. In both bundles, Reward A was blackcurrant juice without or with added monosodium glutamate (MSG), on which the animals sated very little. Reward B was either grape juice, strawberry juice, mango juice, water, apple juice, or grape juice with added inosine monophosphate (IMP) on which the animals sated substantially, or peach juice inducing less satiety than blackcurrant juice. The height of two bars within two visual stimuli indicated the quantities of Reward A and B of each bundle on a horizontal touch monitor (higher bar indicated more reward) (Fig. 1 *A* and *B*). In the Reference Bundle, both rewards were set to specific test quantities. In the Variable Bundle, one reward was set to a current test quantity, and the quantity of the other reward was adjustable.
2) Choice indifference indicates equal subjective value between options. At choice indifference against a constant bundle, a change in subjective value of a reward of the alternative bundle needs to be compensated by a change of the other reward of that alternative bundle.
2a) Specifically, the compensatory change of Reward A of the Variable Bundle required for choice indifference against the constant Reference Bundle was a measure of subjective value loss of Reward B of the Variable Bundle (Fig. 1*C*). The changed IPs of Variable Bundles relative to the constant Reference Bundle are conveniently graphed on a two-dimensional map (Fig. 1*D*). As value loss common to both bundle rewards might not result in IP change, a maintained IP may hide nonselective satiety; therefore, an IP change indicates a value change for a specific reward relative to the other bundle reward, rather than an absolute value change of that specific reward.
3) A series of IPs aligns as an IC. Thus, an IP change that indicates subjective value change results in an IC change (Fig. 1*E*).
4) As consequence of changed IPs and ICs indicating subjective value change, the original, physically unchanged bundles to which the animal was indifferent before satiety fail to match the IPs and ICs established during satiety. The mismatch depends on the reward being sated; it is smaller for less sated rewards and larger for more sated rewards.
5) The mismatch between IPs and ICs established before and during satiety represents the major measure of altered subjective reward value by ongoing consumption and will be used for describing the altered neuronal coding of subjective reward.

### Consumption-Induced Relative Subjective Value Reduction.

A typical test started with a fixed Reference Bundle (0.6 mL blackcurrant juice, no grape juice) and a Variable Bundle (variable blackcurrant juice quantity; fixed 0.3 mL grape juice). We titrated Reward A of the Variable Bundle (common currency) to achieve choice indifference between the two bundles. Psychophysical estimations with Weibull-fitted IPs during blocks of 80 trials demonstrated choice indifference with 0.1 mL blackcurrant juice in the Variable Bundle (Fig. 1*C*, leftmost green IP; *P* = 0.5 choice of each bundle). On a two-dimensional plot of bundle rewards (Fig. 1*D*), the connecting line between two bundles indicated equal subjective value: the Variable Bundle (green dot) had the same subjective value as the Reference Bundle (black dot); the two dots were IPs relative to each other. Thus, at choice indifference, the gain of 0.3 mL grape juice in the Variable Bundle relative to the Reference Bundle had a common-currency value of 0.5 mL blackcurrant juice.

With ongoing consumption of blackcurrant and grape juices, choice indifference required increasing blackcurrant juice, as indicated by successive IPs (Fig. 1*C*, from green via violet, blue, and orange to red at *P* = 0.5). Apparently, the gained 0.3 mL grape juice in the Variable Bundle had lost some of its value for the animal, and only larger blackcurrant juice quantities compensated for that loss. The two IPs within the green 95% confidence interval (CI) suggested initially maintained subjective value of grape juice, whereas continuing consumption moved the next IPs outside the CI, indicating progressive reduction of subjective grape juice value. The consumption-induced rightward IP progression (Fig. 1*C*) translated into an upward movement of IPs on a two-dimensional graph (Fig. 1*D*, from green to red), thus flattening the slope between the two IPs. Thus, the additional blackcurrant juice required for choice indifference provided a common-currency estimate of the subjective value loss of grape juice. Or, compared to the Reference Bundle, the animal gave up progressively less blackcurrant juice to gain the same quantity of grape juice (0.3 mL) (decreased Marginal Rate of Substitution, MRS, of blackcurrant juice for grape juice). These measures indicated increasing subjective value loss of grape juice relative to blackcurrant juice with ongoing consumption.

Wider bundle variations demonstrated consistent consumption-induced subjective value changes (Fig. 1*E*). Starting with a Reference Bundle of 0.6 mL blackcurrant juice alone, the first IP was obtained by setting the Variable Bundle to coordinates inside the *x*–*y* graph. Subsequently, the Reference Bundle was set to the first IP, and another IP was estimated from a newly set Variable Bundle. Repetition of this procedure, in pseudorandomly alternating directions to avoid local distortions (29), resulted in a series of IPs. The curvature of ICs fitted to these IPs (see *Materials and Methods*; Eq. **1**) progressed from convex via near linear to concave (Fig. 1*E**,* green via blue to red). The concavity indicated that the animal required substantial grape juice levels before trading in much blackcurrant juice, possibly reflecting the liquid content of the grape juice, which suggested systematic and advancing subjective value loss of grape juice relative to blackcurrant juice. These slope and curvature changes of two-dimensional IC occurred during recording periods of individual neurons and constituted our test scheme for behavioral and neuronal correlates of reward-specific satiety.

As control, we inverted the test scheme by holding blackcurrant juice constant and varying grape juice psychophysically to obtain choice indifference. Here, instead of the common-currency quantity of blackcurrant juice, the increasing quantity of grape juice at choice indifference quantified its subjective value decrease with ongoing consumption. We used single-reward bundles in which only one, but a different, reward in each bundle had nonzero amount; the consumption of both rewards increased the IPs, flattened the IC slopes, and increased the blackcurrant:grape juice ratios at IPs; when using two-reward bundles, IC curvature changed from convex to concave (*SI Appendix*, Fig. S1 *A*–*D*). The ICs with bundles containing water in Monkey B showed similar slope flattening and reduced convexity with this test scheme (*SI Appendix*, Fig. S1 *E* and *F*). Thus, the consumption-induced relative subjective value reduction of grape juice or water relative to blackcurrant juice was robust irrespective of test scheme.

### Consistency across Bundles.

**Fig. 2.**
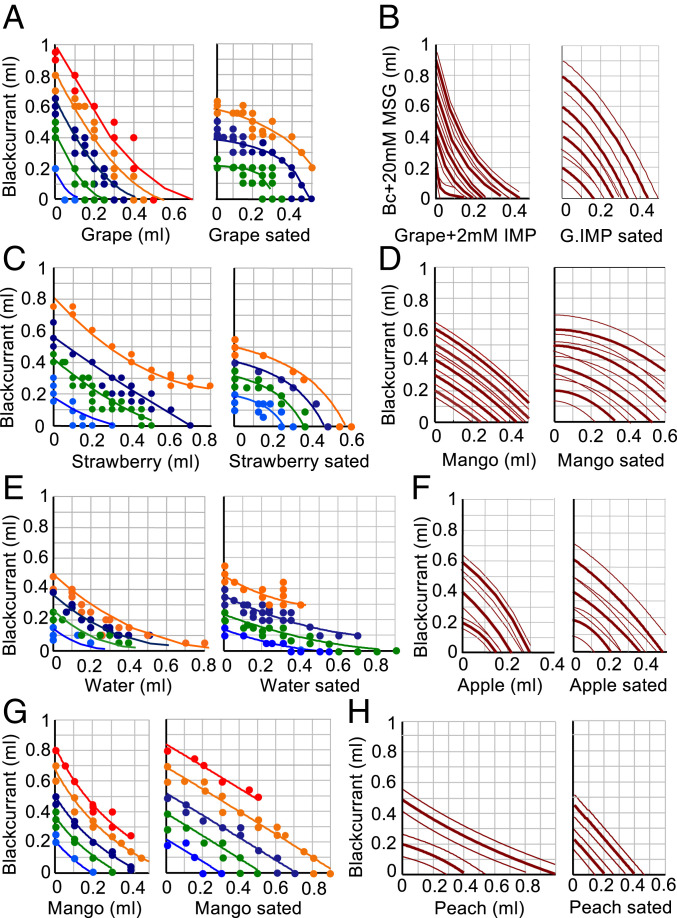
Behavioral effects of reward-specific satiety evidenced by change in choice IC in all bundles tested. (*A*–*F*) Behavioral ICs for all bundle types of the current experiment (Monkey A). Lines show ICs fitted hyperbolically to IP of same color (Eq. **1**). Incomplete lines reflect fitting limited to estimated IPs. Dots in *A, C,* and *E* show measured IPs (choice indifference between all bundles of same color). Thin lines in *B*, *D*, *F*, and *H* show 95% CI. Reward A is plotted on the *y*-axis and Reward B on the *x*-axis. Bc, blackcurrant juice; MSG, monosodium glutamate; IMP, inosine monophosphate. IMP and MSG were only added to the juices shown in *B*. Same color convention in *A*, *C*, *E,* and *G* as in Fig. 1 *C*–*E*. *G* and *H* refer to Monkey B.

Two rhesus monkeys performed 74,659 choices with one or more of the eight bundle types (Fig. 2). We defined the boundary between presated and sated states by the CI of the initial, leftmost choice function between blackcurrant juice and any reward (green in Fig. 1*D* and *SI Appendix*, Fig. S1 *A* and *E*); any IP outside this interval indicated subjective value reduction.

Before satiety, we used a total of 38,443 choices to estimate 56 IPs for fitting up to 5 ICs with the bundle (blackcurrant juice, grape juice), 68 IPs for 4 ICs with bundle (blackcurrant juice, strawberry juice) (Monkey A, unless otherwise noted), 58 IPs for 4 ICs with bundle (blackcurrant juice, water), 38 IPs for 5 ICs with bundle (blackcurrant juice, mango juice) (Monkey B), 65 IPs for 5 ICs with bundle (blackcurrant + MSG, grape + IMP), 55 IPs for 5 ICs with bundle (blackcurrant juice, mango juice), 45 IPs for 3 ICs with bundle (blackcurrant juice, apple juice), and 40 IPs for 2 ICs with bundle (blackcurrant juice, peach juice) (Monkey B).

During satiety, we used 36,216 trials to estimate 52 IPs for 3 ICs with bundle (blackcurrant juice, grape juice), 37 IPs for 4 ICs with bundle (blackcurrant juice, strawberry juice), 63 IPs for 4 ICs with bundle (blackcurrant juice, water), 48 IPs for 5 ICs with bundle (blackcurrant juice, mango juice) (Monkey B), 49 IPs for 4 ICs with bundle (blackcurrant + MSG, grape + IMP), 52 IPs for 4 ICs with bundle (blackcurrant juice, mango juice), 55 IPs for 3 ICs with bundle (blackcurrant juice, apple juice), and 44 IPs for 2 ICs with bundle (blackcurrant juice, peach juice) (Monkey B).

Ongoing reward consumption changed IC curvature from convex to linear and concave or flattened IC slopes, with all bundles tested in this study. The IC change suggested that both animals became more sated across consecutive trials on water, grape juice, strawberry juice, mango juice, and apple juice (*x*-axis) as compared to blackcurrant juice (*y*-axis) (Fig. 2 *A*–*G* and *SI Appendix*, Fig. S1 *D* and *F*–*H*). Nevertheless, the downward slopes of the ICs indicated remaining positive reward value of the sated liquids: increasing amounts of sated liquid (increase on *x*-axis) required less compensatory unsated blackcurrant juice (decrease on *y*-axis) for maintaining choice indifference. None of the sated juices had acquired negative reward value, which would have been indicated by an upward IC slope: increasing amounts of negatively valued sated liquid (increase on *x*-axis) would have required more blackcurrant juice (increase on *y*-axis) for compensation at IC, which was not the case with the currently used liquids but had been detected with lemon juice, yogurt, and saline (25). The only exception to this pattern of satiety was peach juice: IC steepness increased for this bundle, indicating less satiety than for blackcurrant juice (Fig. 2*H*). These IC changes demonstrate robust and systematic relative subjective value changes with natural, ongoing liquid consumption across a variety of bundle types.

### Control for Other Choice Variables.

A logistic regression confirmed that bundle choice varied only with the bundle rewards but not with unrelated variables with ongoing consumption, such as trial number within block of consecutive trials and spatial choice (Eq. **2**). As before satiety (24), the probability of choosing the Variable Bundle continued to correlate positively with the quantities of both its rewards, and inversely with the quantities of both Reference Bundle rewards (*SI Appendix*, Fig. S1*I*; Variable Bundles A and B, VA, VB, versus Reference Bundles A and B, RA, RB). Furthermore, choice probability for the Variable Bundle was anticorrelated with accumulated blackcurrant juice (MA) consumption and positively correlated with grape juice consumption (MB). This asymmetry is explained by the reward quantities at the IPs; as grape juice lost more subjective value than blackcurrant juice during satiety, the animal required, and thus consumed, more grape juice for less blackcurrant juice at the titrated IP. Trial number within individual trial blocks (CT) and spatial choice (CL) did not explain the choice. Thus, even with ongoing consumption, the animals based their choice on the reward quantities of the bundles and the actually consumed rewards; unrelated variables kept having no significant influence.

### Lick Durations.

**Fig. 3.**
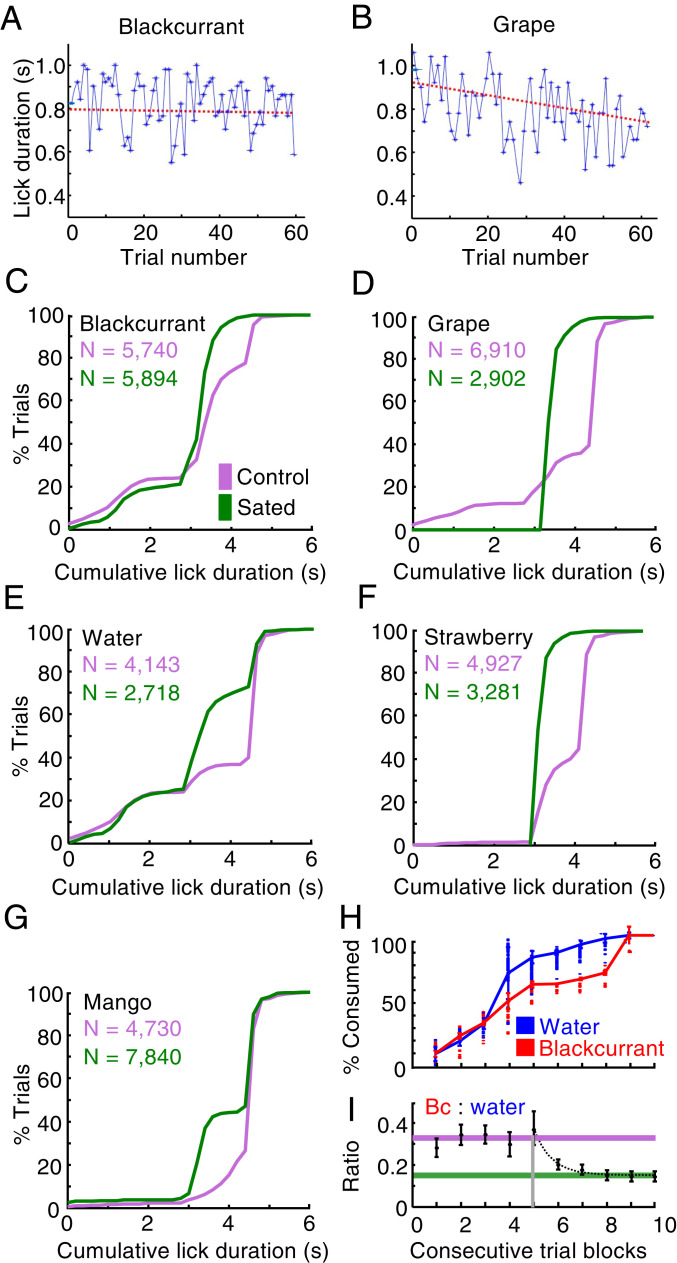
Anticipatory licking and differential juice consumption indicating reward-specific satiety. (*A* and *B*) Differential decrease of anticipatory licking with single-reward bundles during ongoing reward consumption within single test sessions: lick durations remained nearly constant for blackcurrant juice (slope = −2.86°, *R*^2^ = 0.56; 69 trials; linear regression, red) but decreased for grape juice (slope = −20.6°, *R*^2^ = 0.50; 65 trials) (Monkey A). (*C*–*G*) Cumulative distributions of lick durations between bundle appearance and reward delivery for several single-reward bundles. Both animals showed significantly more trials with longer lick durations before (pink) than during satiety (green) where animals engaged less frequently in short licks (*D*). Monkey A, blackcurrant juice: *P* = 5.46 × 10^−4^, Kolmogorov–Smirnov test, *n* = 5,740/5,894 presated/sated trials; grape juice: *P* = 2.59 × 10^−9^, *n* = 6,910/2,902; water: *P* = 3.60 × 10^−3^, *n* = 4,143/2,718; strawberry juice: *P* = 8.66 × 10^−6^, *n* = 4,920/3,281; Monkey B, mango juice: *P* = 2.41 × 10^−9^, *n* = 4,730/7,840. (*H*) Cumulative consumption of water (Reward B) and blackcurrant juice (Reward A) during 10 advancing blocks and 7,160 trials (including bundles with two nonzero quantities). For constant blackcurrant quantities (red), the animal consumed significantly more water than blackcurrant as trials advanced (Monkey A). (*I*) Reduction of blackcurrant:water consumption ratio (Bc:water) from 0.32 (1:3) before satiety (pink) to 0.15 (1:6) with ongoing consumption (green). Single exponential function f (β, x) starting at vertical gray line: β_1_ + β_2_e^(β3x)^; [β_1,_ β_2,_ β_3_] = [0.15, 254.78, −1.41] (β_1_: final ratio, green line; β_2_: decay constant). Consecutive 10 trial blocks for fitting included last block with stable ratio. *n* = 5,520 trials with single-reward bundles; reference *SI Appendix*, Fig. S1 *A* and *B* for test scheme; Monkey A.

Licking provides a simple measure that could serve as mechanism-independent confirmation for the subjective value changes seen with choices. With ongoing reward consumption of both juices, anticipatory licking between bundle stimuli and the first reward changed inconspicuously with blackcurrant juice but decreased gradually with grape juice (Fig. 3 *A* and *B*). This change suggested greater subjective value loss for grape juice than blackcurrant juice. In both animals, cumulative licking varied considerably between the different liquids and decreased significantly during satiety (green) compared to before satiety (pink) (Fig. 3 *C*–*G*). Thus, the licking changes corresponded to the relative reward-specific subjective value changes inferred from bundle choices, IC slopes, and curvatures (Fig. 2).

### Liquid Consumption.

The IC flattening with ongoing consumption indicated that the animal required increasing quantities of the more devalued Reward B for giving up the same quantity of the less devalued blackcurrant juice (Reward A) at IP (Fig. 1*E*). This change was also evident in the choice between the constant Reference Bundle containing only blackcurrant juice (Reward A) and the Variable Bundle containing only one of the other liquids (Reward B) (anchor trials; *SI Appendix*, Fig. S1*B*; increase on *x*-axis). With ongoing consumption, the animal gave up the same quantity of the less sated blackcurrant juice only if it received increasingly more of the sated Reward B at choice indifference. As the animal had no control over the constant Reference Bundle that defined the IP, it ended up consuming more of the devalued reward as the session advanced. For example, with ongoing consumption of the bundle (blackcurrant juice, water), consumption increased more rapidly within each day for water (on which the animal was more sated) than for blackcurrant juice (on which the animal was less sated) (Fig. 3*H*; blue versus red; *P* = 5.0979 × 10^−7^; Kolmogorov–Smirnov test; *n* = 7,160 trials including bundles with two nonzero quantities). The differential consumption change with satiety resulted in decreases of blackcurrant:water consumption ratios at IP (Fig. 3*I*; anchor trials only). These ratio changes indicated relative subjective value changes among the juices (change in common currency).

Similar or less pronounced changes of consumption and consumption ratios occurred with bundles containing blackcurrant juice and grape, mango, or apple juice (*SI Appendix*, Fig. S2 *A*–*D*), corresponding to higher satiety for these juices relative to blackcurrant juice (Fig. 2 *A, D, F,* and *G*). However, both consumption and ratios increased for bundles combining blackcurrant juice with strawberry or peach juice (*SI Appendix*, Fig. S2 *E* and *F*), corresponding to slightly or noticeably higher satiety for blackcurrant relative to strawberry and peach juice (Fig. 2 *C* and *H*). The ratio changes were consistent across successive weekdays while accelerating on Fridays (*SI Appendix*, Fig. S2 *G*–*J*).

To control for the delivery of two different juices, we tested a bundle containing blackcurrant juice as both components and found no change in consumption ratio (*SI Appendix*, Fig. S3*A*). Furthermore, we reversed the juice sequence, delivering grape juice (Reward A) before blackcurrant juice (Reward B), and interchanged their *x–y* coordinates. Ongoing juice consumption consistently changed IC slope and curvature (*SI Appendix*, Fig. S3*B*). The slope became substantially steeper, which corresponded to the flattened slopes with the regular sequence and *x*–*y* coordinates (Fig. 2*A*). The curvature became partly concave, indicating that the animal gave up little grape juice for small blackcurrant juice quantities but much more grape juice for larger blackcurrant juice quantities. Together, these IC changes with reversed juice sequence suggested overall reduction of subjective grape juice value relative to blackcurrant juice that confirmed the satiety in the regular juice sequence and indicated grape juice satiety independent of delivery sequence. Furthermore, temporal consumption profiles were similar for both juices (*SI Appendix*, Fig. S3 *C*, *Top*), whereas consumption ratios reflected well the reduced grape juice value relative to blackcurrant juice (*SI Appendix*, Fig. S3 *C*, *Bottom*) and corresponded to the inverse ratio changes in the opposite, regular juice sequence (*SI Appendix*, Fig. S2*A*).

Thus, the consumption changes confirmed the relative reward-specific subjective value changes inferred from bundle choices.

### Neuronal Test Design.

**Fig. 4.**
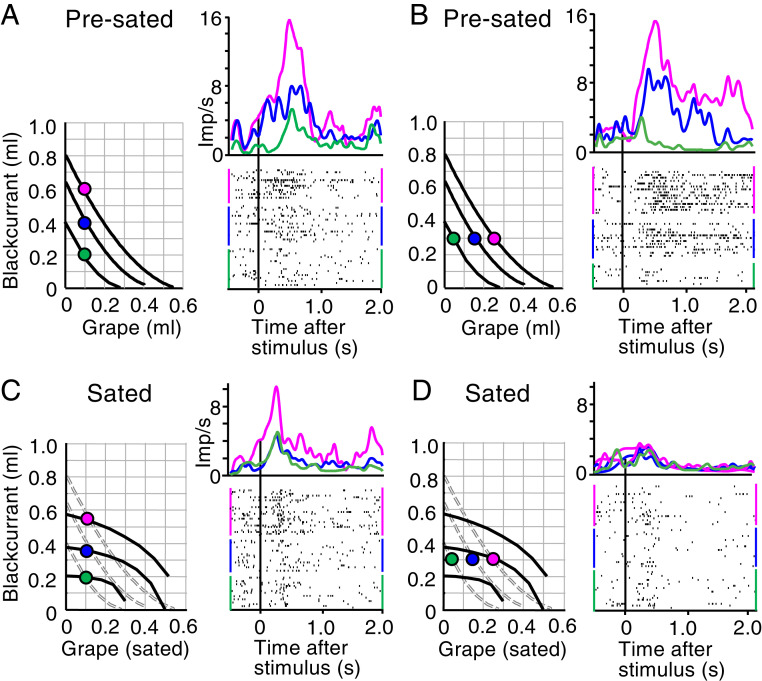
Typical response reduction in single, chosen value coding OFC neuron that follows satiety-related change of choice IC. (*A*) Monotonic response increase across three ICs with increasing blackcurrant juice before satiety during choice over zero-reward bundle (constant grape juice) (*P* = 0.030, *F*(1,35) = 8.86; *P* = 4.3105 × 10^−7^, *F*(2,35) = 15.05; two-way ANOVA: baseline versus poststimulus; across three bundles). Prestimulus activity varied insignificantly across ICs (*P* = 0.6649, *F*(2,14) = 0.41; one-way ANOVA). Binwidth 10 ms, Gaussian kernel smoothing. Each colored dot indicates a bundle with specific blackcurrant and grape juice quantities located on a specific IC. (*B*) Same as *A* but significant response variation with grape juice across ICs (constant blackcurrant juice) (*P* = 3.15305 × 10^−10^, *F*(1,35) = 41.57; *P* = 2.9245 × 10^−24^, *F*(2,35) = 61.9). Insignificant prestimulus variation: *P* = 0.4273, *F*(2, 14) = 0.87. Same colors as *A*. (*C*) After consumption of both bundle rewards while recording from same neuron: only mild effect for blackcurrant juice. Despite IC change, the three bundles remained on their three original and separate ICs, and neuronal coding of blackcurrant juice remained significant (*P* = 0.0275, *F*(1,35) = 4.88; *P* = 5.0096 × 10^−28^, *F*(2,35) = 69.09) but peak response was reduced by 29% (from 15.5 to 11 impulses/s; red) and failed to discriminate between intermediate and low bundles. Insignificant prestimulus variation: *P* = 0.0507, *F*(2,14) = 3.12. Gray dotted lines indicate ICs before satiety, as in *A*. (*D*) Neuronal response change for sated grape juice: response peak reduction by 75% (from 15.2 to 3.8 imp/s; red), and loss of significant variation (*P* = 0.0008, *F*(1,35) = 11.2; *P* = 0.8053, *F*(2,35) = 0.22). Insignificant prestimulus variation: *P* = 0.9686, *F*(2,14) = 0.03. After the consumption-induced slope and curvature change of the ICs (from convex to concave), the three physically unchanged bundles were now on or close to the same, intermediate IC, indicating similar subjective value among them and reflecting satiety for grape juice. Dotted ICs are from presated state. Thus, while continuing to code reward value (*C*), the responses followed the satiety-induced IC change (*D*). This reduction of bundle stimulus response was seen in 30 neurons (*SI Appendix*, Table S1).

Due to the IC change with ongoing reward consumption, the original, physically unchanged bundles that were IPs before satiety failed to match the new ICs estimated during satiety. The mismatch depended on the degree of satiety: bundles with variation of less sated reward showed less mismatch, whereas bundles with variation of more sated reward showed more mismatch. To benefit from the IC scheme, we used two tests: variation of blackcurrant juice while holding grape juice constant, and variation of grape juice while holding blackcurrant juice constant. Comparison of IC maps between the presated state (Fig. 4 *A* and *B*, *Left*) and the sated state (Fig. 4 *C* and *D*, *Left*) shows that IC flattening with satiety moved bundle positions relative to ICs very little for blackcurrant juice variation (Fig. 4 *A* versus *C*) but substantially for grape juice variation (Fig. 4 *B* versus *D*).

We tested the influence of ongoing reward consumption during the recording period of individual neurons, which allowed us to compare responses of the same neuron between nonsated versus sated states, as defined by IPs inside versus outside 95% CI, respectively (Fig. 1*C*, green zone). As these tests required several tens of minutes with each neuron, neurons not coding chosen value were not further investigated. All satiety-tested neuronal responses followed the basic scheme of ICs: monotonic increase with bundles placed on different ICs (testing bundles with different subjective value), and insignificant response variation with bundles positioned along same ICs (testing equally preferred bundles with equal subjective value) (24). We assessed these characteristics with a combination of multiple linear regression (Eq. **3**), Spearman rank correlation, and two-way ANOVA (see *Materials and Methods*). All tested responses belonged to the subgroup of previously studied OFC neurons (30) that were sensitive to multiple rewards and coded the value of the bundle the animal chose (“chosen value,” as defined by Eqs. **4** and **5**). To reduce visual confounds between the two choice options, we used similar visual stimuli that were not identifiable as distinct objects; therefore, we could not test object value or offer value coding as the other major OFC response category (1).

We subjected most neurons to two bundle tests: 1) choice over zero-reward bundle, both rewards were set to zero in one bundle, and the animal unfailingly chose the alternative, nonzero bundle; and 2) choice between two nonzero bundles, at least one reward was set to nonzero in both bundles, and the animal chose either bundle (Table 1). In addition, for comparison with previous studies on single-reward options, we tested all neurons with single-reward bundles in which only one reward was set to nonzero; usually the nonzero reward differed between the two bundles.

**Table 1.** Numbers of neurons tested with ongoing reward consumption

Bundle type	Choice over zero-reward bundle		Choice between two nonzero bundles	
	Neurons tested	IPs tested	Neurons tested	IPs tested
Blackcurrant, grape	21 + 11 = 32	28	7 + 12 = 19	38
Blackcurrant, water	20 + 13 = 33	39	22 + 12 = 34	58
Blackcurrant, mango	14 + 7 = 21	11	9 + 8 = 17	10
SUM	55 + 31 = 86	78	38 + 32 = 70	106

The bundle types (blackcurrant, grape) and (blackcurrant, water) were tested in Monkey A (81 and 138 neurons, respectively), whereas bundle type (blackcurrant, mango) was tested in Monkey B (53 neurons). All neurons stated coded chosen value, as identified by Eqs. **4** and **5**, and followed the IC scheme defined previously (30): monotonic increase or monotonic decrease with bundles compared across ICs, insignificant response variation with bundles compared along individual ICs. Such neurons were recorded only during choice over zero-reward bundle (*n* = 28 neurons), only during choice between two nonzero bundles (*n* = 12 neurons), or both (*n* = 58 neurons) (total of 98 neurons). In table cells with multiple entries, the first two numbers refer respectively to positive and negative (inverse) relationships to increasing reward quantity, as inferred from the neuronal regression slope (β's in Eq. **3**). IP; bundle at choice indifference point at specific *x*–*y* coordinate.

### Single-Neuron Subjective Value Coding Follows IC Changes.

At the beginning of daily testing, neuronal responses during choice over zero-reward bundle followed monotonically the increase of both bundle rewards (blackcurrant and grape juice), confirming value coding. Stimuli for bundles on higher ICs elicited significantly larger responses (Fig. 4 *A* and *B*) (prestimulus increases due to the short intertrial interval varied insignificantly and were unrelated to trial sequence; *SI Appendix*, Fig. S4). With ongoing consumption of both bundle rewards, increasing blackcurrant juice, on which the animal was less sated, remained positioned on different ICs. Correspondingly, neuronal responses continued to vary across ICs (here, primarily between the top two ICs) (Fig. 4*C*; red versus blue-green). By contrast, increasing grape juice did not require much compensation by decrease of blackcurrant juice for maintaining choice indifference, as shown by the flattened and concave ICs (Fig. 4 *D*, *Left*). The IC change indicated that more grape juice had only little more reward value to the animal. Accordingly, the neuron recorded during this test showed only a small and unmodulated response despite grape juice increase (Fig. 4 *D*, *Right*; *P* = 0.22; across-bundle factor of two-way ANOVA); the response peak for the largest grape juice quantity dropped by 75%. Thus, ongoing consumption of both juices reduced the subjective value variation of grape juice, and the neuronal responses reflected the reduced value variation, without a change in coding rules or reward selectivity.

Similar consumption-induced neuronal changes occurred in choice between two nonzero bundles (*SI Appendix*, Fig. S5). As bundles varying only in blackcurrant juice continued to occupy different ICs, OFC responses continued to increase with blackcurrant juice (*SI Appendix*, Fig. S5 *A* and *C*). By contrast, as original bundles varying only in grape juice were now positioned on lower and fewer ICs, neuronal responses decreased and became less differential (*SI Appendix*, Fig. S2 *B* and *D*; red, blue, green). Responses to the physically unchanged bundle whose position had changed from intermediate to highest IC (hollow blue) now dominated all other responses (*SI Appendix*, Fig. S5 *D*, *Right*, dotted blue line). Finally, before satiety the bundle containing only 0.6 mL blackcurrant juice had similar subjective value as the bundle with only 0.4 mL grape juice (*SI Appendix*, Fig. S5*B*; hollow and solid blue dots on same IC) and correspondingly drew similar neuronal responses (dotted and solid blue lines), whereas with satiety the same two bundles were positioned on different ICs (*SI Appendix*, Fig. S5*D*; hollow versus solid blue dot) and correspondingly elicited different responses (dotted versus solid blue line). Thus, the neuronal responses changed with ongoing reward consumption irrespective of choice over zero-reward bundle or true choice between two nonzero bundles.

Thus, OFC neurons continued to code reward value with ongoing reward consumption. The responses continued to discriminate well the quantity of blackcurrant juice whose subjective value had changed relatively less (Fig. 4 *A* versus *C* and *SI Appendix*, Fig. S5 *A* versus *C*) but were reduced for grape juice whose value had dropped more, despite unchanged physical grape juice quantities (Fig. 4 *B* versus *D* and *SI Appendix*, Fig. S5 *B* versus *D*). In following the altered ICs, these OFC signals reflected reward-specific relative subjective value changes induced by ongoing consumption.

### Neuronal Population.

Out of 424 tested OFC neurons, 272 showed changes during task performance and were investigated during ongoing reward consumption neurons. These neurons were located in area 13 at 30 to 38 mm anterior to the interaural line and lateral 0 to 19 mm from the midline; they were parts of the population reported previously (30). Responses in 98 of these 272 task-related OFC neurons (36%) coded chosen value (defined by Eqs. **4** and **5**) and followed the IC scheme in any of the four task epochs (Bundle stimulus, Go, Choice, or Reward) during choice over zero-reward bundle or choice between two nonzero bundles (Table 1). Of the 98 chosen value neurons, 82 showed satiety-related changes with bundles composed of blackcurrant juice (Reward A) and grape juice, water, or mango juice (Reward B) (Table 2 and *SI Appendix*, Table S1).

**Table 2.** Satiety-induced neuronal changes

	Neurons tested	Response decreases during satiety		Response increases during satiety		No satiety effects
		Neurons	Responses	Neurons	Responses	Neurons
Choice over zero-reward bundle						
Positive coding	55	31	101	21	69	3
Negative coding	31	14	33	15	54	2
Choice between two nonzero bundles						
Positive coding	38	16	54	16	57	6
Negative coding	32	14	36	9	31	9

The table includes data from chosen value responses from all task epochs (Bundle stimulus, Go, Choice, or Reward) and all bundles tested for satiety (Reward A: blackcurrant juice, Reward B: grape juice, water, or mango juice).

**Fig. 5.**
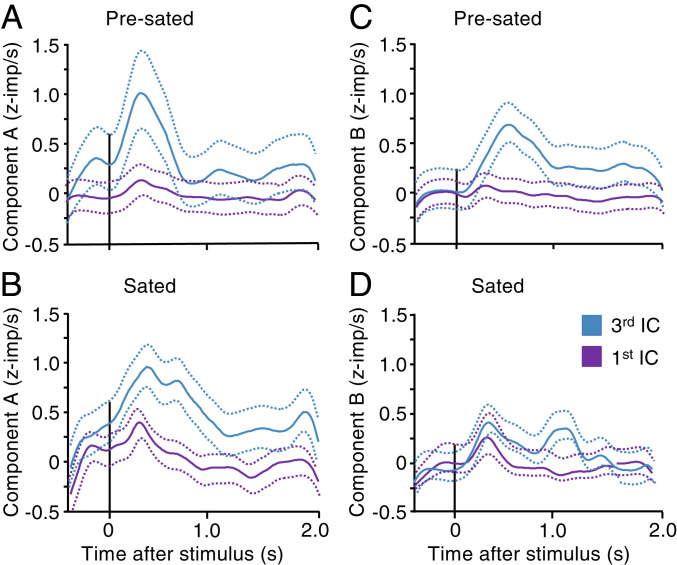
Reduction of neuronal population response with reward-specific satiety. (*A*–*D*) Differential satiety-related reduction of averaged *z*-scored chosen value responses in 30 positive coding neurons from both animals. Each panel shows responses and their 95% CI to bundles on lowest and third lowest IC during choice over zero-reward bundle. For Reward A (blackcurrant juice), responses differed significantly between lowest and third lowest ICs both before satiety (*A*: *P* = 0.0015, *F*(1,3650) = 10.12; *P* = 5.0564 × 10^−4^, *F*(1,3650) = 19.7; two-way ANOVA: baseline versus poststimulus; across two bundles) and during satiety (*B*: *P* = 3.10864 × 10^−5^, *F*(1,3853) = 17.39; *P* = 1.35252 × 10^−32^, *F*(1,3853) = 143.99). By contrast, for Reward B (grape juice, water, or mango juice), responses differed significantly before satiety (*C*: *P* = 0.0005, *F*(1,5769) = 12.22; *P* = 9.54232 × 10^−10^, *F*(1,5769) = 38.97) but not during satiety (*D*: *P* = 0.0028, *F*(1,4790) = 9.65; *P* = 0.91, *F*(1,4790) = 0.01).

Using the scheme of differential consumption-induced IC changes of Fig. 4 and *SI Appendix*, Fig. S5, we found that averages of *z*-scored positive subjective value coding responses in 31 neurons (Table 2) continued to vary with blackcurrant juice quantity (despite peak reduction) (Reward A; Fig. 5 *A* and *B*), whereas responses became insignificant with grape juice, water, or mango juice in the same neurons (43% peak reduction) (Reward B; Fig. 5 *C* and *D*). These contrasting, consumption-related neuronal response variations occurred individually in all four task epochs (*SI Appendix*, Table S1).

Quantifications of individual response changes demonstrated consumption-induced significant response reduction with positive subjective value coding neurons and significant response increase with negative (inverse) coding neurons (lower response with higher subjective value) during choice over zero-reward bundle (*SI Appendix*, Fig. S6 *A* and *B*, red) and during choice between two nonzero bundles (*SI Appendix*, Fig. S6 *C* and *D*, red; Table 2 and *SI Appendix*, Table S1). Fewer neurons showed inverse changes that were difficult to reconcile with subjective value coding (black in *SI Appendix*, Fig. S6) or insignificant changes.

Thus, OFC neuronal population responses reflected consumption-induced changes in subjective value in a similar way as individual neurons.

### Neuronal Classifier Performance.

We used a neuronal classifier as additional means for demonstrating changes of neuronal reward coding by ongoing reward consumption. We analyzed only neuronal responses that followed the IC scheme; they increased or decreased monotonically across ICs during any of the four task epochs but changed only insignificantly along ICs; furthermore, the responses changed with ongoing reward consumption as shown in Figs. 4 and 5 and *SI Appendix*, Fig. S5.

**Fig. 6.**
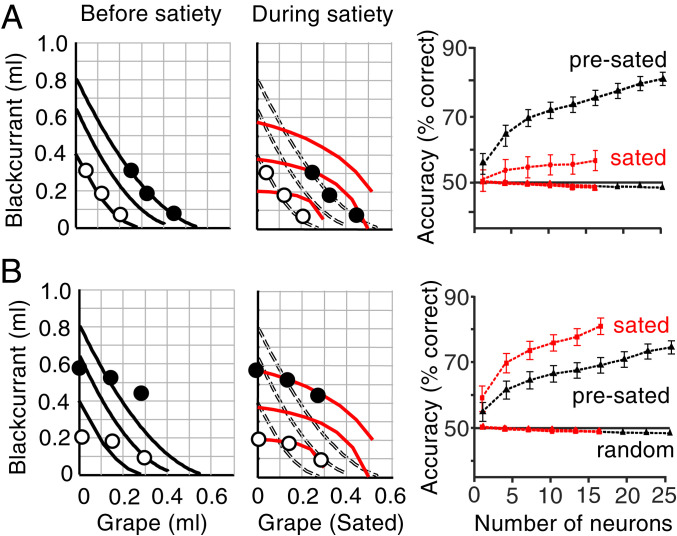
Bundle classification demonstrates satiety-related change of neuronal subjective value coding. (*A*) Bundle classification by support vector machine using neuronal responses to stimuli of bundles positioned on the lowest and third lowest indifference curve, respectively (choice over zero-reward bundle; Bundle stimulus epoch). The classifier was trained on neuronal responses collected before satiety and tested for bundle distinction before satiety (black) and during satiety (red). (*Left*) Identical bundle positions on two-dimensional map but IC change with ongoing consumption, indicating satiety-induced relative subjective value change (red). (*Right*) Classifier accuracy increase with neuron numbers before satiety (black) but drop when tested during satiety (red). Error bars indicate SEMs. Random: control classification with shuffled assignment of neuronal responses to tested ICs. (*B*) Same as *A* but reverse testing order: classifier trained on neuronal responses collected during satiety and tested during satiety (red) and before satiety (black).

We first established the accuracy with which an ideal observer using neuronal responses distinguished bundles on different ICs. We trained a support vector machine classifier on neuronal responses to randomly selected bundles positioned on the lowest and highest of three ICs (Bundle stimulus epoch). Before satiety, as defined by IPs inside the initial CI (Fig. 1*C*), the classifier showed decent bundle discrimination with as few as five neurons during choice over zero-reward bundle; classifier performance increased meaningfully with added neurons (Fig. 6*A*, black). Then, we used this classifier trained on presatiety responses and tested distinction of the same bundles using neuronal responses during consumption-induced satiety. We found a significant difference of accuracy (*F*(1, 34) = 68.02, *P* = 1.26653 × 10^−9^; one-way ANOVA); the accuracy increase with added neurons suggested maintained valid classification (Fig. 6*A*, red). The satiety-related accuracy drop was also evident in the inverse test sequence: whereas accuracy was high with classifier training and testing during satiety, it was significantly lower when training on neuronal responses during satiety but testing using responses before satiety (*F*(1, 34) = 17.99, *P* = 0.0002).

The change in classifier accuracy occurred with choice over zero-reward bundle with neuronal responses to Bundle stimuli (Fig. 6) and during the Go epoch (*SI Appendix*, Fig. S7*A*) but not during Choice and Reward epochs (*SI Appendix*, Fig. S7 *B* and *C*). The changes were not explained by baseline changes during the 1-s pretrial control epoch (*SI Appendix*, Fig. S7*D*). Similar accuracy differences were seen in choice between two nonzero bundles during Bundle stimuli, Go epoch, and Choice epoch but not during the Reward epoch (*SI Appendix*, Fig. S7 *E*–*H*), again unexplained by baseline changes (*SI Appendix*, Fig. S7*I*). The accuracy differences were consistent across ongoing consumption steps (*SI Appendix*, Fig. S7*J*).

Finally, we analyzed activity from 265 unmodulated and unselected neurons, as before (27). From the total of 424 tested OFC neurons, we excluded the 98 neurons whose activity followed the IC scheme and a further 61 neurons that coded only one of the two bundle rewards. Of the remaining 265 neurons, 113 showed task-related activity, whereas the other 152 neurons did not. The classification from the activities of the 265 neurons still showed mild differences between presatiety training responses and satiety test responses during the task epochs of Bundle stimuli, Go, and Choice (*SI Appendix*, Figs. S8 and S9). These remaining differences may reflect subsignificance changes and suggest that subjective value coding reflects satiety mildly in a rather large OFC population.

Thus, the classifier results provide additional evidence for neuronal responses reflecting consumption-induced subjective value changes indicative of satiety.

### Neuronal Changes with Single-Reward Bundles.

Our use of choice options with two reward components differs from the conventional use of single-reward options (1, 2) and thus requires controls and additional analyses. To do so, we used the same two visual component stimuli but set only one, but different, reward in each bundle to a nonzero quantity. The reward settings allowing choice indifference were derived from our use of two-reward bundles. The single-reward bundles were positioned graphically along the *x*-axis and *y*-axis but not inside the IC map (*SI Appendix*, Fig. S1*B*) and thus were equivalent to single-reward choice options tested earlier (1, 3, 26). Trials with these anchor bundles interleaved with trials using two regular bundles of which at least one contained two nonzero rewards. These regular bundles provided the IPs at the axes required for analyzing anchor bundle choices.

**Fig. 7.**
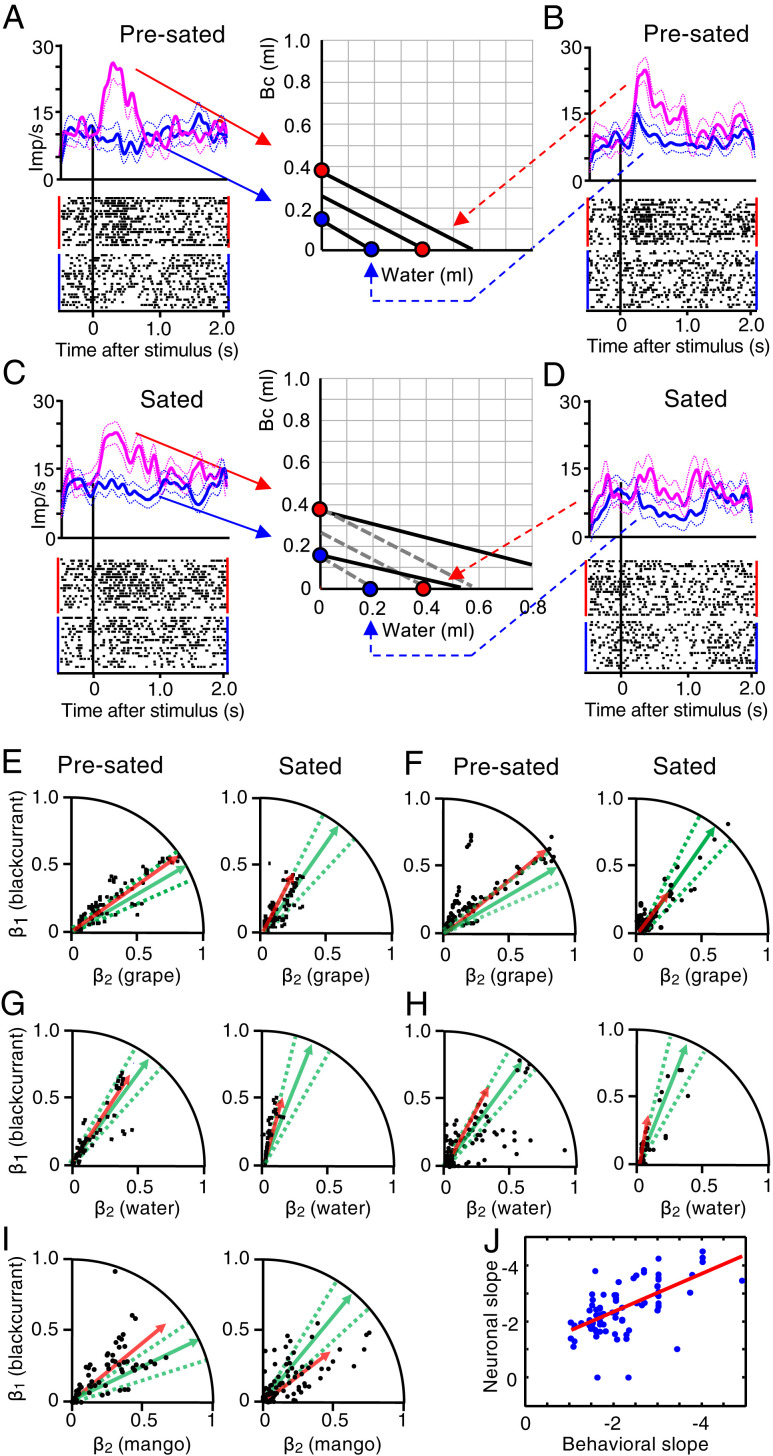
Reward-specific satiety confirmed with single-reward bundles. (*A*–*D*) Responses of one chosen value coding neuron before and during satiety. Each bundle contained specific nonzero quantities of only blackcurrant juice or only water (colored dots on *x*- and *y*-axes) and was tested during choice over zero-reward bundle. ICs were obtained from IPs estimated in interleaved trials with two-reward bundles. Binwidth 10 ms, Gaussian kernel smoothing, 95% CI. (*A*) Significant response increase across two ICs with increasing blackcurrant juice (Bc) before satiety (water remained zero) (*P* = 9.54232 × 10^−10^, *F*(1,40) = 38.97; *P* = 8.3368 × 10^−10^, *F*(1,40) = 39.27; two-way ANOVA: baseline versus poststimulus; red versus blue bundle). (*B*) Same as *A* but significant response variation with increasing water across two ICs (blackcurrant juice remained zero) (*P* = 9.8258 × 10^−6^, *F*(1,40) = 20.02; *P* = 2.0472 × 10^−8^, *F*(1,40) = 32.54). Same colors as *A*. (*C*) Despite IC flattening with ongoing reward consumption, the two bundles with blackcurrant juice variation remained on the same two ICs, and the neuronal response variation remained significant (*P* = 4.7616 × 10^−8^, *F*(1,40) = 30.91; *P* = 6.9739 × 10^−13^, *F*(1,40) = 54.5), with some peak response reduction (red). Dotted ICs are from presated state. (*D*) IC flattening with reward consumption indicates relative subjective value reduction of water. The two unchanged bundles with water variation were now located on and below the lower IC (dotted lines). Neuronal activity varied only weakly (red) (*P* = 8.9470 × 10^−8^, *F*(1,40) = 29.6; *P* = 0.0367, *F*(1,40) = 4.39). Furthermore, the large-water bundle (dotted red line) now elicited a similar response as the low-blackcurrant bundle that was now on the same IC (solid blue line in *C*). Thus, while continuing to code subjective reward value (shown in *C*), the responses followed the satiety-induced IC change. This reduction of bundle stimulus response was seen in 30 neurons (*SI Appendix*, Table S1). (*E*) Vector plots for behavioral choice of bundle (blackcurrant juice, grape juice) over zero-reward bundle (green) and corresponding *z*-scored neuronal population responses (rectified for inverse coding) of all 32 tested chosen value neurons, unselected for satiety change (black, red). Neuronal vector slopes were 35° before satiety and 62° during satiety, pooling all significantly positive and normalized negative (inverse) coding responses from all four task epochs (Tables 1 and 2); all included responses followed the IC scheme. Dots refer to neuronal responses, vectors represent averages from behavioral choices (green; dotted lines: 95% CI) and neuronal responses (red), based on Eqs. **1a** and **3**, respectively (see *Materials and Methods*). Neuronal slope regression coefficients (β’s) on axes refer to Eq. **3**. (*F*) Same as *E* but for choice between two nonzero bundles. Neuronal vector slopes were 38° before and 45° during satiety. (*G* and *H*) Same as *E* and *F* but for bundle (blackcurrant juice, water) (*n* = 33 unselected chosen value neurons). (*I*) Same as *E* but for bundle (blackcurrant juice, mango juice) (*n* = 21 unselected chosen value neurons). The larger deviation between behavioral (green) and neuronal (red) vectors for mango juice (*I*) compared to grape juice and water (*E*–*H*) may be due to fewer neurons tested and fewer tests (Table 1). (*J*) Correlation between rectified neuronal and behavioral IC slopes (β’s from Eqs. **3** and **1a**, respectively) during satiety in all tested neurons (rho = 0.604; *P* = 8 × 10^−6^, Pearson correlation; rho = 0.595, *P* = 2 × 10^−5^, Spearman rank correlation; *n* = 90 responses during choice between two nonzero bundles).

First, we confirmed the results from two-reward bundles using the same IC formalism. We tested single-reward bundles on the same 82 neurons that had shown satiety-related changes with two-reward bundles. The neuronal responses of Fig. 7 *A* and *B* distinguished both blackcurrant juice and water quantities during choice over zero-reward bundle before satiety. Ongoing consumption of both rewards flattened the ICs. The IC relationship of blackcurrant juice was preserved, and the neuron kept discriminating blackcurrant juice quantities (Fig. 7*C*). By contrast, the large water quantity was now positioned further below the top IC than before (Fig. 7*D*, red on *x*-axis) and close to the IC of the small blackcurrant quantity (blue on *y*-axis), and the small water quantity was now positioned below its original IC (blue on *x*-axis). Correspondingly, neuronal activity with large water quantity lost its peak and varied only weakly between the two water quantities (Fig. 7*D*, blue versus red). These neuronal changes with single-reward bundles reflected the consumption-induced subjective value changes in a similar way as with two-reward bundles (Fig. 4 and *SI Appendix*, Fig. S5).

Next, we used single-reward bundles to compare neuronal response changes with behavioral changes. We established vector plots that displayed the ratio of reward weights (β's) for *z*-scored neuronal population responses (Eq. **3**; Fig. 7 *E*–*I*, red) and, separately, for behavioral choice (Eq. **1a**; green). The inequality of subjective value between the two rewards was manifested as deviation of these vectors from the 45° diagonal line. Ongoing reward consumption increased the elevation angle of the behavioral vector, indicating subjective value loss for Reward B (grape juice, water, or mango juice) relative to Reward A (blackcurrant juice). The neuronal vector changed correspondingly (Fig. 7 *E*–*I*, red). For example, during choice of the bundle (blackcurrant juice, grape juice) over zero-reward bundle, the behavioral vector angle increased from 40° before satiety to 65° during satiety, and the neuronal population vector increased from 35° to 62° (Fig. 7*E*, green, red). Similarly, during choice between two nonzero bundles, the behavioral vector increased from 40° to 52° while the neuronal vector increased from 38° to 45° (Fig. 7*F*). Furthermore, the shorter neuronal vectors during satiety indicated general reduced responding, indicating additional general satiety (red). Bundles containing water or mango juice showed similar changes (Fig. 7 *G*–*I*). Thus, both before and during satiety, the neuronal vectors (red) were within the CIs of the behavioral vectors (green), indicating intact neuronal subjective value coding that reflected the value changes with ongoing reward consumption.

In addition to the vector analysis, IC slopes confirmed the close neuronal–behavioral correspondence during satiety, with satiety being defined by IPs exceeding the initial, presated psychophysical CIs (*SI Appendix*, Fig. S1 *A* and *E*). As estimated from regression coefficient ratios (−β_2_/β_1_) (Eq. **3**) and (−b/a) (Eq. **1**), the slopes of the linear neuronal ICs of single-reward bundles correlated with the slopes of linear behavioral ICs (Fig. 7*J*). These results from single-reward bundles during ongoing reward consumption compared well with the results from the earlier OFC study on single rewards with spontaneously varying subjective reward value (1).

Taken together, the consumption-induced changes in single OFC neurons and in the OFC population corresponded well between single-reward and two-reward choice options. This similarity provides a sound foundation for the current findings on the more natural multicomponent choice options.

## Discussion

This study investigated whether the reward responses of OFC neurons reflected reductions of subjective economic reward value induced by satiety for specific rewards, using stringent tests based on formal concepts of economic decision theory. As reward-specific satiety constitutes a major contributor to subjective value, the observed changes present crucial support in favor of economic value coding in OFC neurons and extend the role of this cortical structure in economic decision mechanisms. The use of bundles containing two differently sated rewards assured choice of both options as essential requirement for assessing subjective reward value in a controlled and unequivocal manner at choice indifference. The value loss was captured by graphic ICs that were constructed from psychophysically estimated IPs and represented the subjective economic value of the bundles. The ICs changed in an orderly and characteristic manner with ongoing reward consumption (Figs. 1 and 2 and *SI Appendix*, Fig. S1); they flattened progressively and changed from convex via near linear to concave, thus indicating gradual subjective value loss for all bundle rewards (plotted on the *x*-axis) relative to blackcurrant juice (*y*-axis) (except for lesser peach juice value loss). These IC changes suggested that the animal required increasing quantities of the less sated blackcurrant juice for compensating the increasing subjective value loss of the other, more sated bundle reward with ongoing consumption of both rewards. The specific and asymmetric IC changes made alternative explanations unlikely, such as general satiety, motivation loss, passage of time, or proximity of return to home cage, all of which would affect all rewards nondifferentially. Licking behavior and consumption supported the notion of reward-specific satiety in a mechanism-independent manner (Fig. 3 and *SI Appendix*, Figs. S2 and S3). Taken together, the demonstrated sensitivity to reward-specific satiety contributes an important argument in favor of formal subjective economic value coding by OFC neurons inferred from other choice data.

Our preceding study had established neuronal chosen value responses in OFC that were sensitive to multiple rewards and followed the animal's rational choice of two-reward bundles, including completeness, transitivity, and independence from option set size (24). The current study tested the effects of ongoing reward consumption on these chosen value responses. We found that the neuronal responses matched the consumption-induced IC changes during recording periods of individual neurons. The responses became weaker for more sated rewards (Figs. 4 and 5 and *SI Appendix*, Figs. S4 and S5). Most impressively, neuronal responses failed to distinguish between bundles that were physically unchanged but came to be on the same ICs because of the ICs’ curvature change to concave (Fig. 4*D*). Classifiers predicting bundle discrimination from neuronal responses confirmed accurate subjective reward value coding both before and during satiety and demonstrated the substantial nature of neuronal changes (Fig. 6 and *SI Appendix*, Figs. S7–S9). Neuronal response vectors of conventional single-reward choice options correlated well with behavioral choice vectors; the correlations were maintained during consumption-induced subjective value changes (Fig. 7). These results demonstrate that OFC responses followed the differential subjective value changes induced by ongoing reward consumption. As the rewards did not change physically with satiety, these results satisfy a necessary requirement for the coding of subjective value by OFC neurons that had been suggested based on choices between single-reward options (1) and two-reward bundles (30).

The consumption increase of sated rewards like water, grape juice, apple juice, and mango juice (Fig. 3*H* and *SI Appendix*, Fig. S2 *A*–*D*) seemed to contradict earlier findings and the general intuition that satiety would rather reduce consumption of rewards on which an animal is sated. Differences in study design might explain these discrepancies. In the earlier studies, monkeys chose between sated and nonsated rewards, or between accepting and not accepting sated rewards, and naturally preferred nonsated rewards (8, 9, 15–17). As this tendency precluded choice indifference, we studied choice between bundles that each contained two differently sated rewards. Advancing satiety on Reward B required increasing quantities of this reward for choice indifference against bundles containing the less sated Reward A (Fig. 1*E* and *SI Appendix*, Fig. S1*B*). Thus, satiety increased consumption of the sated reward. By contrast, outright rejection of the more sated Reward B would have been represented by an upward sloped IC, which had been observed with lemon juice, yogurt, and saline (24) but not with the currently used rewards; such upward sloped ICs indicated that an animal needed to be “bribed” with more reward for accepting these normally rejected sated rewards. By contrast, in the current study, the maintained downward IC slope indicated that the animal was not entirely averse to the sated reward.

The currently reported altered neuronal value coding with reward-specific satiety builds on previous studies on monkey OFC neurons that investigated satiety by clinical tests and behavioral observations. There, monkeys were presented with syringes or tubes containing various fruit juices; rating scales and go–no-go lick responses indicated behavioral acceptance or rejection of these juices (8, 9). The studies report that OFC neurons lost responses only for the particularly sated juice. Spontaneous variations of single-reward choices likely reflected appetite variations over the course of daily experimentation and affected chosen value coding in monkey OFC (1). Stronger general satiety effects in ventromedial prefrontal cortex compared to OFC (11) suggest widespread satiety sensitivity in ventral frontal cortex. Human studies using pleasantness ratings demonstrate neuroimaging correlates of reward satiety in OFC (12–14). The presence of satiety effects in OFC contrast with their absence in the earlier gustatory system, including nucleus of the solitary tract, frontal opercular taste cortex, and insular taste cortex (6, 7, 31). The neurophysiological results (1, 8, 9, 11) correspond to the reward functions of frontal cortex that become deficient after lesion and inactivation, including associative strength (or, equivalently, incentive value), cognitive reward representation, and approach and goal-directed behavior (10, 15–17). Our present data on subjective value coding in a specific choice context extend these results into the domain of economic decision-making.

In addition to reward-specific satiety, ongoing consumption also induces general satiety that may consist of a general loss of pleasure, arousal, attention, and motivation. Our shorter neuronal population vectors with single-reward bundles may reflect general satiety, in addition to the changed vector angle that suggests reward-specific satiety (Fig. 7 *E*–*I*, red). General and unspecific satiety would lead to general subjective value reduction in a similar way as reduced physical reward quantity; both would be represented by parallel, or curvi-parallel, IC displacement toward the *x*–*y* graph origin (Fig. 2 *A*–*H*, *Left*), whereas reward-specific satiety is indicated by changes in IC slope and curvature (*Right*). General satiety is seen with reduced midbrain responses in humans during consumption of Swiss chocolate (12) and reduced dopamine responses in mice receiving food pellets for extended periods of time (32). Thus, general satiety cannot explain our asymmetric IC changes and the corresponding asymmetric neuronal changes, both of which reflect subjective value changes indicative of reward-specific satiety.

## Materials and Methods

The study used the same two male adult rhesus monkeys as previously (24, 30) and was licensed by the UK Home Office (for details, reference *SI Appendix*). The animals chose between two compound stimuli that were positioned at pseudorandomly alternating fixed left–right positions on a computer monitor and predicted bundles containing the same two rewards whose quantities varied pseudorandomly and without specific temporal order. We psychophysically estimated multiple choice IPs (Fig. 1*C* and *SI Appendix*, Fig. S1 *A* and *E*) to which we fitted ICs along which all bundles were equally preferred, using a hyperbolic function *d*:

d=ay+bx+cxy, [1]

with *y* and *x* as milliliter quantity of Rewards A and B (Fig. 1 *D* and *E* and *SI Appendix*, Fig. S1 *B*, *D*, and *F*), *a* and *b* as weights of the influence of the two reward quantities, and *c* as curvature. Eq. **1** can be equivalently rewritten as regression in analogy to the regression used for analyzing neuronal responses:

$$y=\beta_{0}+\beta_{1}A+\phi_{2}B+\beta_{3}AB+\varepsilon ,$$ [1a]

with *A* and *B* as milliliter quantity of Reward A (plotted at *y*-axis) and Reward B (*x*-axis), respectively, β_0_ as offset coefficient, β_1_ and β_2_ as behavioral regression coefficients, and ε as compound of errors *err*_0_, *err*_1_, *err*_2_, and *err*_3_ for offset and regressors 1 to 3.

To test whether the animal’s choice reflected the quantity of the bundle rewards during satiety, rather than other, unintended variables such as spatial bias, we used the following logistic regression:

$$P\left(V\right)=\beta_{0}+\beta_{1}CT+\beta_{2}RA+\beta_{3}RB+\beta_{4}VA+\beta_{5}VB+\beta_{6}CL+\beta_{7}MA+\beta_{8}MB+\varepsilon ,$$ [2]

with *P* (*V*) as probability of choice of Variable Bundle, β_0_ as offset coefficient, β_1_ to β_7_ as correlation strength (regression slope) coefficients indicating the influence of the respective regressor, *CT* as trial number within blocks of consecutive trials, *RA* as quantity of Reward A of Reference Bundle, *RB* as quantity of Reward B of Reference Bundle, *VA* as quantity of Reward A of Variable Bundle, *VB* as quantity of Reward B of Variable Bundle, *CL* as choice of any bundle stimulus presented at the left, *MA* as consumed quantity of Reward A, *MB* as consumed quantity of Reward B, and ε as compound error for offset and all regressors.

Following behavioral training and surgical preparation for single-neuron recording, we identified neuronal task relationships with the paired Wilcoxon test. We identified changes of task-related neuronal responses across ICs with a linear regression:

$$y=\beta_{0}+\beta_{1}A+\beta_{2}B+\beta_{3}AB+\varepsilon ,$$ [3]

with *y* as neuronal response in any of the four task epochs (Bundle stimulus, Go, Choice, Reward), measured as impulses/s and *z*-scored normalized to the pretrial control epoch of 1.0 s, *A* and *B* as milliliter quantity of Reward A (plotted at *y*-axis) and Reward B (*x*-axis), respectively, *β*_*0*_ as offset coefficient, *β*_*1*_ and *β*_*2*_ as neuronal regression coefficients, and ε as compound error. In addition, all significant neuronal response changes across ICs identified by Eq. **3** needed to be also significant in a Spearman rank-correlation test (*P* < 0.05).

To assess neuronal compliance with the two-dimensional IC scheme, we used a two-factor ANOVA on each task-related response that was significant for both regressors in Eq. **3**. Neuronal responses following the IC scheme were significant across ICs (factor 1: *P* < 0.05) but insignificant within IC (factor 2).

Chosen value (*CV*) was defined as follows:

$$CV=A+k_{1}B,$$ [4]

weighting parameter *k*_1_ served to adjust for differences in subjective value between Rewards A and B, such that the quantity of Reward B entered the regression on a common-currency scale defined by Reward A. We assessed neuronal coding of chosen value in all neurons that followed the revealed preference scheme, using the following regression:

$$y=\beta_{0}+\beta_{1}CV+\beta_{2}UCV+\varepsilon ,$$ [5]

with *UCV* as value of the unchosen option that was not further considered here, and ε as compound error.

## Supplementary Material

Supplementary File

## Data Availability

All study data are included in the article and/or *SI Appendix*.

## References

[1] C. Padoa-Schioppa, J. A. Assad, Neurons in the orbitofrontal cortex encode economic value. Nature 441, 223–226 (2006). 16633341 10.1038/nature04676 PMC2630027

[2] L. Tremblay, W. Schultz, Relative reward preference in primate orbitofrontal cortex. Nature 398, 704–708 (1999). 10227292 10.1038/19525

[3] S. Kobayashi, W. Schultz, Influence of reward delays on responses of dopamine neurons. J. Neurosci. 28, 7837–7846 (2008). 18667616 10.1523/JNEUROSCI.1600-08.2008 PMC3844811

[4] M. Cabanac, Physiological role of pleasure. Science 173, 1103–1107 (1971). 5098954 10.1126/science.173.4002.1103

[5] E. T. Rolls, B. J. Rolls, E. A. Rowe, Sensory-specific and motivation-specific satiety for the sight and taste of food and water in man. Physiol. Behav. 30, 185–192 (1983). 6844432 10.1016/0031-9384(83)90003-3

[6] S. Yaxley, E. T. Rolls, Z. J. Sienkiewicz, T. R. Scott, Satiety does not affect gustatory activity in the nucleus of the solitary tract of the alert monkey. Brain Res. 347, 85–93 (1985). 4052808 10.1016/0006-8993(85)90891-1

[7] E. T. Rolls, T. R. Scott, Z. J. Sienkiewicz, S. Yaxley, The responsiveness of neurones in the frontal opercular gustatory cortex of the macaque monkey is independent of hunger. J. Physiol. 397, 1–12 (1988). 3411507 10.1113/jphysiol.1988.sp016984 PMC1192108

[8] E. T. Rolls, Z. J. Sienkiewicz, S. Yaxley, Hunger modulates the responses to gustatory stimuli of single neurons in the caudolateral orbitofrontal cortex of the macaque monkey. Eur. J. Neurosci. 1, 53–60 (1989). 12106174 10.1111/j.1460-9568.1989.tb00774.x

[9] H. D. Critchley, E. T. Rolls, Olfactory neuronal responses in the primate orbitofrontal cortex: Analysis in an olfactory discrimination task. J. Neurophysiol. 75, 1659–1672 (1996). 8727404 10.1152/jn.1996.75.4.1659

[10] B. W. Balleine, A. Dickinson, Goal-directed instrumental action: Contingency and incentive learning and their cortical substrates. Neuropharmacology 37, 407–419 (1998). 9704982 10.1016/s0028-3908(98)00033-1

[11] S. Bouret, B. J. Richmond, Ventromedial and orbital prefrontal neurons differentially encode internally and externally driven motivational values in monkeys. J. Neurosci. 30, 8591–8601 (2010). 20573905 10.1523/JNEUROSCI.0049-10.2010 PMC2942083

[12] D. M. Small, R. J. Zatorre, A. Dagher, A. C. Evans, M. Jones-Gotman, Changes in brain activity related to eating chocolate: From pleasure to aversion. Brain 124, 1720–1733 (2001). 11522575 10.1093/brain/124.9.1720

[13] M. L. Kringelbach, J. O’Doherty, E. T. Rolls, C. Andrews, Activation of the human orbitofrontal cortex to a liquid food stimulus is correlated with its subjective pleasantness. Cereb. Cortex 13, 1064–1071 (2003). 12967923 10.1093/cercor/13.10.1064

[14] J. A. Gottfried, J. O’Doherty, R. J. Dolan, Encoding predictive reward value in human amygdala and orbitofrontal cortex. Science 301, 1104–1107 (2003). 12934011 10.1126/science.1087919

[15] A. Izquierdo, R. K. Suda, E. A. Murray, Bilateral orbital prefrontal cortex lesions in rhesus monkeys disrupt choices guided by both reward value and reward contingency. J. Neurosci. 24, 7540–7548 (2004). 15329401 10.1523/JNEUROSCI.1921-04.2004 PMC6729636

[16] P. H. Rudebeck, R. C. Saunders, A. T. Prescott, L. S. Chau, E. A. Murray, Prefrontal mechanisms of behavioral flexibility, emotion regulation and value updating. Nat. Neurosci. 16, 1140–1145 (2013). 23792944 10.1038/nn.3440 PMC3733248

[17] E. A. Murray, E. J. Moylan, K. S. Saleem, B. M. Basile, J. Turchi, Specialized areas for value updating and goal selection in the primate orbitofrontal cortex. eLife 4, e11695 (2015). 26673891 10.7554/eLife.11695 PMC4739757

[18] J. Von Neumann, O. Morgenstern, The Theory of Games and Economic Behavior (Princeton University Press, Princeton, NJ, 1944).

[19] P. A. Samuelson, A note on measurement of utility. Rev. Econ. Stud. 4, 155–161 (1937).

[20] A. Mas-Colell, M. Whinston, J. R. Green Microeconomic Theory (Oxford University Press, New York, 1995).

[21] W. Schultz, Neuronal reward and decision signals: From theories to data. Physiol. Rev. 95, 853–951 (2015). 26109341 10.1152/physrev.00023.2014 PMC4491543

[22] M. L. Platt, P. W. Glimcher, Neural correlates of decision variables in parietal cortex. Nature 400, 233–238 (1999). 10421364 10.1038/22268

[23] D. J. Barraclough, M. L. Conroy, D. Lee, Prefrontal cortex and decision making in a mixed-strategy game. Nat. Neurosci. 7, 404–410 (2004). 15004564 10.1038/nn1209

[24] A. Pastor-Bernier, C. R. Plott, W. Schultz, Monkeys choose as if maximizing utility compatible with basic principles of revealed preference theory. Proc. Natl. Acad. Sci. U.S.A. 114, E1766–E1775 (2017). 28202727 10.1073/pnas.1612010114 PMC5347590

[25] C. Frydman, C. Camerer, P. Bossaerts, A. Rangel, MAOA-L carriers are better at making optimal financial decisions under risk. Proc. Biol. Sci. 278, 2053–2059 (2011). 21147794 10.1098/rspb.2010.2304 PMC3107654

[26] W. R. Stauffer, A. Lak, W. Schultz, Dopamine reward prediction error responses reflect marginal utility. Curr. Biol. 24, 2491–2500 (2014). 25283778 10.1016/j.cub.2014.08.064 PMC4228052

[27] I. Fisher, Mathematical investigations in the theory of value and prices. Trans. Connecticut Acad. 9, 1–124 (1892).

[28] P. A. Samuelson, A note on the pure theory of consumer’s behavior. Economica 5, 61–71 (1938).

[29] J. L. Knetsch, The endowment effect and evidence of nonreversible indifference curves. Am. Econ. Rev. 79, 1277–1288 (1989).

[30] A. Pastor-Bernier, A. Stasiak, W. Schultz, Orbitofrontal signals for two-component choice options comply with indifference curves of Revealed Preference Theory. Nat. Commun. 10, 4885 (2019). 31653852 10.1038/s41467-019-12792-4 PMC6814743

[31] S. Yaxley, E. T. Rolls, Z. J. Sienkiewicz, The responsiveness of neurons in the insular gustatory cortex of the macaque monkey is independent of hunger. Physiol. Behav. 42, 223–229 (1988). 3406148 10.1016/0031-9384(88)90074-1

[32] M. A. Rossi, D. Fan, J. W. Barter, H. H. Yin, Bidirectional modulation of substantia nigra activity by motivational state. PLoS One 8, e71598 (2013). 23936522 10.1371/journal.pone.0071598 PMC3735640

