# Supplementary material for: Reward-specific satiety affects subjective value signals in orbitofrontal cortex during multicomponent economic choice
Source: Proc Natl Acad Sci U S A. 2021 Jul 20;118(30):e2022650118. doi: 10.1073/pnas.2022650118 (PMC8325167; doi:10.1073/pnas.2022650118)
Supplement: Supplementary File [file pnas.2022650118.sapp.pdf]

Supplementary information for

Reward-specific satiety affects subjective value signals in orbitofrontal cortex during multi-component economic choice

Alexandre Pastor-Bernier, Arkadiusz Stasiak and Wolfram Schultz*

This PDF file includes:

Supplementary Methods
Figures S1 to S9
Table S1

Supplementary Methods

Animals. Two adult male macaque monkeys (*Macaca mulatta*; Monkey A, Monkey B), weighing 11.0 kg and 10.0 kg, respectively, were used in these experiments that had already yielded behavioral and neuronal data without satiety (1, 2). Neither animal had been used in any other study.

Ethical approval. This research has been ethically reviewed, approved, regulated and supervised by the following institutions and individuals in the UK and at the University of Cambridge (UCam): the Minister of State at the UK Home Office, the Animals in Science Regulation Unit (ASRU) of the UK Home Office implementing the Animals (Scientific Procedures) Act 1986 with Amendment Regulations 2012, the UK Animals in Science Committee (ASC), the local UK Home Office Inspector, the UK National Centre for Replacement, Refinement and Reduction of Animal Experiments (NC3Rs), the UCam Animal Welfare and Ethical Review Body (AWERB), the UCam Governance and Strategy Committee, the Home Office Establishment License Holder of the UCam Biomedical Service (UBS), the UBS Director for Governance and Welfare, the UBS Named Information and Compliance Support Officer, the UBS Named Veterinary Surgeon (NVS), and the UBS Named Animal Care and Welfare Officer (NACWO).

General behavior. The animals were habituated during several months to sit in a primate chair (Crist Instruments) for a few hours each working day. They were trained in a specific, computer-controlled behavioral task in which they contacted visual stimuli on a horizontally mounted touch-sensitive computer monitor (Elo) located 30 cm in front of them. The animal's eye position in the horizontal and vertical plane were monitored with a non-invasive infrared oculometer (Iscan). Matlab software (Mathworks) running on a Microsoft Windows XP computer controlled the behavior and collected, analyzed and presented the data on-line. A solenoid valve (ASCO, SCB262C068) controlled by the same Windows computer served to deliver specific liquid quantities. A Microsoft SQL Server 2008 Database served for Matlab off-line data analysis. Following task training for about 6 months, animals were surgically implanted with a recording chamber for electrophysiological recordings, which typically lasted for another 6-10 months.

Stimuli and rewards. A computer touch monitor presented the subject with two visual stimuli (4 deg apart), each representing one choice option, called Reference Bundle and Variable Bundle (Fig. 1*A*). Each bundle contained two rewards that were represented separately by a colored rectangle: Reward A, represented by a violet rectangle, and Reward B, represented by a green rectangle. Reward quantities were set independently and indicated by the vertical position of a bar within each rectangle (higher was more). The Reference Bundle contained two preset Reward quantities that were fixed for a given block of trials. The Variable Bundle contained a specifically set quantity of one Reward and an experimentally varied quantity of the other reward. Reward A in all bundles was blackcurrant juice without or with added monosodium glutamate (MSG), Reward B was grape juice, strawberry juice, mango juice, water, apple juice, peach juice, or grape juice with

added inosine monophosphate (IMG). While MSG and IMG are known taste enhancers, we did not test choice preferences between them on their own. Thus, we cannot state whether they had own reward value for the animals.

Task. Each trial began when the animal contacted a centrally located touch sensitive key for 1.0 s after a pseudorandom inter-trial interval of 1.6 ± 0.25 s. Then the two stimulus pairs representing the two bundles appeared at pseudorandomly alternating fixed left-right positions on a computer monitor in front of the animal (Fig. 1*B*). After 2.0 s, two blue spots appeared as GO stimulus underneath the bundle stimuli, upon which the animal released the touch key and touched the blue spot underneath the bundle of its choice within 2.0 s. The required action consisted of one arm movement and was constant across bundles and trials. After a hold time of 1.0 s, the blue spot underneath the chosen bundle turned green, and the blue spot underneath the unchosen bundle disappeared. Simultaneously a white frame around the chosen bundle appeared as feedback for successful choice. The computer-controlled liquid solenoid valve delivered Reward A at 1.0 s after the choice, followed 0.5 s later by Reward B (except when using peach juice as Reward B; here the sequence was reversed: Reward B was delivered first, then 0.5 s later Reward A, blackcurrant juice). Task training was initially restricted to one bundle type and was extended to other bundle types only when satisfactory behavioral performance was obtained.

The longer delay for liquid B compared to liquid A likely generated asymmetric temporal discounting that affected the subjective value of each liquid. However, all delays were kept constant, which allowed the subjective value differences from different temporal discounting to be incorporated as a constant factor into the subjective value of each liquid. We choose this delay, rather than simultaneous delivery or pseudorandomly alternating single liquid delivery, to prevent more serious taste interactions between simultaneously delivered liquids and to avoid temporal uncertainty. Reaching for a target before appearance of the blue dots, or key release during required key touch or target-hold, were considered as errors and lead directly to the inter-trial interval without reward.

Estimation of behavioral ICs. The behavioral method for obtaining an IP from stochastic choice has been presented in full detail (1, 2). With two bundle options, the animal chose between the pre-set Reference Bundle (left in Fig. 1*A*) and the Variable Bundle (right) in repeated trials. Thus, the constant Reference Bundle provided a stable reference against the changing bundle composition in the Variable Bundle. We set one reward in the Variable Bundle to one unit (≥ 0.1 ml) above the quantity of the same reward in the Reference Bundle, while pseudorandomly varying the quantity of the other reward of the Variable Bundle over the whole test range and in pseudorandom temporal order within each block of trials. The variation of the animal's repeated choice with that single, pseudorandomly varying reward allowed us to construct a full psychophysical function and estimate an IP from Weibull fitting (point of subjective equivalence; $P = 0.5$ choice of each bundle).

As in our previous study (1), we used the Matlab function GLMFIT for psychophysical fitting. This function returns a number called 'Deviance' between 0 and infinity that can be used to compare fitting between Weibull and logit. The Deviance is the difference between the log-likelihood of the fitted model and the maximum possible log-likelihood. Lower values are better. The estimated Deviance for psychophysics for the first 5,000 trials and 2 monkeys was 1.0415 for the Weibull model and 1.6009 for the logit model, suggesting that the Weibull fitted the data better. Hence, we used Weibull fitting for all psychophysical fitting.

We obtained each IP from a total of 80 trials (2 left-right stimulus positions with 5 equally spaced reward quantities in 8 trials). To avoid known adaptations in OFC neurons (3-6), we always tested the full reward range of the experiment.

To obtain an IC, we fit a series of IPs with a hyperbolic function d using weighted least mean squares:

$$d = ay + bx + cxy \qquad \textbf{[1]}$$

with y and x as milliliter quantity of Reward A (plotted on y-axis on 2D graph, Fig. 1*D*, *E*) and Reward B (plotted on x-axis), *a* and *b* as weights of the influence of the Reward quantities plotted on the y- and x-axes, respectively, and *c* as curvature. A potent reward that contributes strongly to the choice of the bundle would have a large weight (high coefficient a or b), whereas a less potent reward would have lower weight coefficients. Thus, with the potent (more weight) reward plotted on the x-axis, and the less potent (less weight) reward plotted on the y-axis, choice indifference between them would occur with smaller milliliter quantities on the x-axis compared to the y-axis. Hence, the IC slope would be steeper than the diagonal line (see Fig. 1*D, E*). By resolving Eq. **1** as $y = -(b / a) * x$, the IC slope would be the ratio of the coefficients that reflect the weights of the rewards: $-b / a$. With a higher potency of Reward B (x-axis) compared to Reward A (y-axis), the rectified IC slope would be larger than 1. Relatively stronger satiety for Reward B (x-axis) compared to Reward A (y-axis) would reduce the weight of Reward B, reduce the absolute value of the ratio $-b / a$, and flatten the IC slope. Thus, the IC slope $-b$ / a describes the relative impact of the two bundle rewards (reflecting the value ratio between the two rewards), whereas the weights (*a* and *b*) describe the influence of the reward quantities.

The hyperbolic function can be re-written in an equivalent form to the regression with interaction used for analysing neuronal responses (see Eq. **3** below):

$$y = \beta_0 + \beta_1 A + \beta_2 B + \beta_3 AB + \varepsilon \qquad \textbf{[1a]}$$

with *A* and *B* as milliliter quantity of Reward A (plotted at y-axis) and Reward B (x-axis), respectively, β_0 as offset coefficient, β_1 and β_2 as behavioral regression coefficients, and ε as compound of errors err_0, err_1, err_2, err_3 for offset and regressors 1-3.

Definition and criteria for pre-sated and sated states. With on-going reward consumption, the changes of psychophysical choice functions exceeding the confidence intervals (CI) of initial tests suggested a changed subjective value relationship between the two bundle rewards suggestive of relative, reward-specific satiety (see Figs. 1*D*, S1*A*, S1*E*). More specifically, the gradual effect of satiety on choice preference was identified by tracking the IPs as consumption advanced across blocks of 80 trials. Importantly, these changes occurred fast enough to be studied during the recording durations of single neurons, thus allowing us to compare responses between non-sated and sated states in the same neuron. The Weibull-fitted IPs were obtained psychophysically for fixed and equally spaced quantities of Reward B. Changes in relative subjective value of the two bundle rewards were assessed with interleaved anchor trials in choices between bundles with only one non-zero reward: bundle (fixed non-zero blackcurrant juice; no Reward B) vs. bundle (no blackcurrant juice; variable non-zero Reward B), using any Reward B (Fig. S1*B*). To aggregate IP data across sessions and compensate for across-session variability, we normalized the reward quantity ratio to the first titration block in all sessions. We then compared the normalized distributions of IPs within the CI of the first block with the distributions of IPs exceeding the CI of the first block.

Control regression for behavioral choice. To test whether the animal's choice reflected the quantity of the bundle rewards during satiety, rather than other, unintended variables such as spatial bias, we used the logistic regression

$$P\,(\mathrm{V}) = \beta_0 + \beta_1 CT + \beta_2 RA + \beta_3 RB + \beta_4 VA + \beta_5 VB + \beta_6 CL + \beta_7 MA + \beta_8 MB + \varepsilon \qquad \textbf{[2]}$$

with *P* (V) as probability of choice of Variable Bundle, β_0 as offset coefficient, β_1 - β_7 as correlation strength (regression slope) coefficients indicating the influence of the respective regressor, *CT* as trial number within blocks of consecutive trials, R*A* as quantity of Reward A of Reference Bundle, *RB* as quantity of Reward B of Reference Bundle, VA as quantity of Reward A of Variable Bundle, *VB* as quantity of Reward B of Variable Bundle, *CL* as choice of any bundle stimulus presented at the left, MA as consumed quantity of Reward A, *MB* as consumed quantity of Reward B, and ε as compound error for offset and all regressors. We used a binomial fit with logit

link function to obtain standardized β coefficients. Choices over zero-reward bundles were excluded in the regression to avoid internal correlation between value and consumption.

Licking. Licking was monitored with an infrared optosensor positioned below the juice spout (V6AP; STM Sensors). Anticipatory lick durations were measured between the appearance of the bundle stimuli and delivery of the first reward liquid (approximately 5 - 6 s duration) in bundles containing only one non-zero reward (single-reward bundles) within single working sessions. Licking data were collected with four bundle types, namely (blackcurrant juice, grape juice), (blackcurrant juice, water), (blackcurrant juice, strawberry juice) and (blackcurrant juice, mango juice).

Surgical procedures and electrophysiology. As described before for the same animals (2), a head-restraining device and a recording chamber (40 x 40 mm, Gray Matter) were implanted on the skull under full general anesthesia and aseptic conditions. The stereotactic coordinates of the chamber enabled neuronal recordings of the orbitofrontal cortex (OFC) (7). We located the OFC from bone marks on coronal and sagittal radiographs taken with a guide cannula inserted at a known coordinate in reference to the implanted chamber, using a medio-lateral vertical and a 20° degree forward-directed approach aiming for area 13. Monkey A provided data from the left hemisphere and Monkey B from the right hemisphere via a craniotomy ranging from Anterior 30 to Anterior 38 and Lateral from 0 to 19. We conducted single-neuron electrophysiological recordings using both custom made glass-coated tungsten electrodes (8) and commercial electrodes (Alpha Omega) (impedance of about 1 MOhm at 1 kHz). Electrodes were inserted into the cortex with a multi-electrode drive (NaN drive) with the same angled approach as used for the radiography. Neuronal signals were collected at 20 kHz, amplified using conventional differential amplifiers (CED 1902 Cambridge Electronics Design) and band-passed filtered (high: 300 Hz, low: 5 kHz). We used a Schmitt-trigger to digitize the analog neuronal signal online into a computer-compatible TTL signal. However, we did not use the Schmitt-trigger to separate simultaneous recordings from multiple neurons, in which case we searched for another recording from only a single neuron, or we stored occasionally the data in analog form for off-line separation by dedicated software (Plexon offline sorter). An infrared eye tracking system monitored eye position (ETL200; ISCAN), with temperature check on an experimenter's hand at the approximate position of the animal's head.

Definition of neurons following the revealed preference scheme. We analysed single-neuron activity during four task epochs vs. Pretrial control (1 s): visual Bundle stimulus (2 s), Go signal (1 s), Choice (1 s) and Reward (2 s, starting with Reward A, followed 0.5 s later by Reward B, except where noted, thus covering both rewards). To establish neuronal relationships to these task epochs, we compared the activity in each neuron during each task epoch separately against the Pretrial control epoch using the paired Wilcoxon test ($P < 0.01$). A neuron was considered task-related if its activity in at least one of the four task epochs differed significantly from the activity during the Pretrial control epoch.

Responses of individual neurons should follow the scheme of two-dimensional ICs that characterizes revealed behavioral preferences for two-dimensional bundles. Specifically, the responses should comply with three characteristics defined previously (2).

(Characteristic 1) Neuronal responses should change monotonically with increasing behavioral preference *across behavioral ICs*, irrespective to bundle composition. Such monotonic neuronal response changes should reflect increasing quantities of one or both bundle rewards, assuming a positive monotonic subjective value function on reward quantity.

(Characteristic 2) Neuronal responses should vary insignificantly for all equally preferred bundles positioned *along a same behavioral IC*, despite different physical bundle composition.

(Characteristic 3) Neuronal responses should follow the IC slope and the nonlinear curvature of behavioral ICs. The IC slope reflects the subjective value relationship between the two bundle rewards, and thus the subjective value of one reward (in our case Reward B) in the common currency of a reference reward (Reward A).

We used a combination of three statistical tests to assess these characteristics.

Characteristic 1: To capture the change *across ICs* in the most conservative, assumption-free manner possible, we used a simple linear regression on each Wilcoxon-identified task-related response:

$$y = \beta_0 + \beta_1 A + \beta_2 B + \beta_3 AB + \varepsilon \quad \textbf{[3]}$$

with y as neuronal response in any of the four task epochs, measured as impulses/s and z-scored normalized to the Pretrial control epoch of 1.0 s (z-scoring of neuronal responses applied to all regressions listed below), A and B as milliliter quantity of Reward A (plotted at y-axis) and Reward B (x-axis), respectively, β_0 as offset coefficient, β_1 and β_2 as neuronal regression coefficients, and ε as compound error for offset and all regressors.

The coefficients β_1 and β_2 needed to be either both positive (indicating positive neuronal relationship, higher neuronal activity reflecting more reward quantity) or both negative (inverse neuronal relationship) to reflect the additive nature of the individual bundle components giving rise to revealed preference ($P < 0.05$, unless otherwise stated; t-test).

This linear regression assessed the degree of linear monotonicity of neuronal response change across ICs ($P < 0.05$ for β coefficients; t-test). Further, all significant positive or negative response changes identified by Eq. **3** needed to be also significant in a Spearman rank-correlation test that assessed ordinal monotonicity of response change across ICs without assuming linearity and numeric scale ($P < 0.05$).

Characteristics 1 and 2: To assess the two-dimensional *across/along IC* scheme in a direct and intuitive way, and without assuming monotonicity, linearity and numeric scale, we used a two-factor Anova on each Wilcoxon-identified task-related response that was also significant for both regressors in Eq. **3**; the factors were *across-IC* (ascending rank order of behavioral ICs) and *along-IC* (same rank order of behavioral IC). To be a candidate for following the IC scheme of Revealed Preference Theory, changes across-ICs should be significant ($P < 0.05$), changes within-IC should be insignificant, and their interaction should be insignificant.

Characteristic 3: Whereas the regression defined by Eq. **3** estimated neuronal responses across ICs, a full estimation of neuronal ICs for comparison with behavioral ICs would require inclusion of the IC slope and curvature, both of which depended on both rewards. By simplifying Eq. **3** by setting to zero both the β_3 coefficient and the constant neuronal response along the IC, the neuronal IC slope would be the ratio of coefficients ($-\beta_2 / \beta_1$). Note the different meanings of the slope term: the neuronal IC slope ($-\beta_2 / \beta_1$) describes the relative coding strength of the two bundle rewards (reflecting the neuronal ratio of the two rewards), whereas each neuronal regression slope alone (β) describes the coding strength of neuronal response (correlation with the specific regressor). The neuronal IC curvature was estimated from the β_3 coefficient of the interaction term AB (all β's $P < 0.05$; t-test).

Neuronal chosen value coding. As stated before (2), chosen value (CV) was defined as the value of the option the animal was going to choose or had already chosen. As each option consisted of two components, we used a linear combination of the quantity of Reward A (blackcurrant juice) and Reward B (any of the other five rewards):

$$CV = A + k_1 B \quad \textbf{[4]}$$

Weighting parameter k_1 served to adjust for differences in subjective value between rewards A and B. We established parameter k_1 during neuronal recording sessions from behavioral choice IPs using quantitative psychophysics in anchor trials (80 trials per test, see above Trial types for neuronal tests), rather than reading it from fitted ICs. Thus, k_1 equals the ratio of coefficients β_2 / β_1 of Eq. 3.

We established a common-currency scale in ml for all tested rewards by defining blackcurrant juice or blackcurrant-MSG (Reward A) as reference (numeraire); the subjective value of any reward is expressed as real-number multiple k_1 of the quantity of the numeraire at choice indifference.

Specifically, the animal chose between the Variable Bundle that contained a psychophysically varied quantity of blackcurrant juice and the Reference Bundle that contained a fixed quantity of blackcurrant juice. A k_1 of < 1 indicated that more quantity was required for choice indifference against blackcurrant juice; thus, $k_1 < 1$ suggested that the tested reward had lower subjective value than blackcurrant juice. By contrast, $k_1 > 1$ suggested higher subjective value, as less quantity was required for choice indifference.

We assessed the coding of chosen value and unchosen value in all neurons that followed the revealed preference scheme, using the following regression:

$$y = \beta_0 + \beta_1 CV + \beta_2 UCV + \varepsilon \qquad \textbf{[5]}$$

with *UCV* as value of the unchosen option that was not further considered here, and ε as compound error for offset and all regressors.

Vector plots of OFC reward sensitivity. The purpose of this analysis was to provide quantitative and graphic information about satiety-induced behavioral and neuronal changes that would allow comparison with previous OFC studies that had not used two-component choice options with individually varying reward quantities and therefore did not establish ICs (9). This simplified analysis addressed monotonic response increase or decrease with increasing quantities of bundle rewards across ICs (characteristic 1 above), but did not address other IC characteristics such as trade-off, slope and curvature (characteristics 2 and 3) that had not been investigated previously. We established 2D plots whose dots indicated the relative contribution of each of the two bundle rewards to the neuronal response. We then compared vectors of behavioral choices with vectors of averaged neuronal population responses before and during satiety.

For behavioral choices, we plotted vectors (with 95% confidence intervals) from averaged dot positions defined by reward quantity (distance from center: sqrt ($\beta_1^2 + \beta_2^2$)) and relative weight (elevation angle: arctangent (β_1 / β_2)); coefficient β_1 refers to Reward A (blackcurrant, y-axis), coefficient β_2 refers to any of the other rewards (x-axis) (Eq. **1a**). The angle of the vector reflects the relative contribution the two bundle rewards to the choice, as estimated by the a and b coefficients (Eq. **1**). A deviation of the alignment angle from the diagonal line indicates an unequal contribution weight to bundle choice, and thus a non-1:1 reward ratio.

For neuronal responses, each dot on the two-dimensional plot was defined by the two β regression coefficients for neuronal responses (Eq. **3**; $P < 0.01$, t-test) for each of the two rewards in any of the four task epochs. The distance from center indicates the z-scored response magnitude (sqrt ($\beta_1^2 + \beta_2^2$)), coding sign (positive or negative), and relative weight (elevation angle; arctangent (β_1 / β_2)) of the two β coefficients. Coefficient β_1 refers to Reward A (blackcurrant, y-axis), coefficient β_2 refers to any of the other rewards (x-axis). Responses with negative (inverse) coding were rectified. Further IC characteristics such as systematic trade-off across multiple IPs and IC curvature played no role in these graphs. The alignment of the dots along the diagonal axis shows the relative coding strength for the two bundle rewards, as estimated by the β regression coefficients; a deviation from the diagonal line indicates an unequal influence of the two bundle rewards on the neuronal responses, reflecting a neuronal correlate of reward ratio.

Neuronal decoding. We used a linear support vector machine (SVM) classifier to decode neuronal activity according to bundles presented at different behavioral ICs during choice over zero-reward bundle (bundle distinction) and, separately, according to the behavioral choice between two non-zero bundles located on different ICs (choice prediction). As in our main study on revealed preferences (2), we implemented the decoder with linear kernel using custom-written software with svmtrain and svmclassify procedures in Matlab R2015b (Mathworks). (our previous work had shown that use of nonlinear SVM kernels did not improve decoding) (10). The SVM decoder was trained to find the optimal linear hyperplane for the best separation between two neuronal populations relative to lower vs. higher ICs.

All analyses employed single-neuron data, consisting of single-trial impulse counts that had been z-normalised to the activity during the Pretrial epoch in all trials recorded with the neuron

under study. The analysis included activity from all neurons whose responses followed the IC scheme of revealed preferences during any of the four task epochs, as identified by our three-test statistics, except where noted. The neurons were recorded one at a time; therefore, the analysis concerned aggregated pseudo-populations of neuronal responses.

The decoding analysis used 10 trials per neuron for each of two ICs (total of 20 trials). Extensive analysis suggested that higher inclusion of 15-20 trials per group did not provide significantly better decoding rates (while reducing the number of included neurons). For neurons that had been recorded with > 10 trials per IC, we selected randomly 10 trials from each neuron for each of the two ICs. We used a leave-one-out cross-validation method in which we removed one of the 20 trials and trained the SVM decoder on the remaining 19 trials. We then used the SVM decoder to assess whether it accurately detected the IC of the left-out trial. We repeated this procedure 20 times, every time leaving out another one of the 20 trials. These 20 repetitions resulted in a percentage of accurate decoding (% out of n = 20). The final percentage estimate of accurate decoding resulted from averaging the results from 150 iterations of this 20-trial random selection procedure. To distinguish from chance decoding, we randomly shuffled the assignment of neuronal responses to the tested ICs, which should result in chance decoding (accuracy of 50% correct). A significant decoding with the real, non-shuffled data would be expressed as statistically significant difference against the shuffled data ($P < 0.01$; Wilcoxon rank-sum test).

SI References

1. A. Pastor-Bernier, C. R. Plott, W. Schultz, W Monkeys choose as if maximizing utility compatible with basic principles of revealed preference theory. *Proc. Natl. Acad. Sci. U. S. A.* **114**, E1766-E1775, (2017).
2. A. Pastor-Bernier, A. Stasiak, W. Schultz, Orbitofrontal signals for two-component choice options comply with indifference curves of Revealed Preference Theory. *Nat. Comm.* **10**, 4885 (2019).
3. L. Tremblay, W. Schultz, Relative reward preference in primate orbitofrontal cortex. *Nature* **398**, 704–708 (1999).
4. C. Padoa-Schioppa, Range-adapting representation of economic value in the orbitofrontal cortex. *J. Neurosci.* **29**, 14004-14014 (2009).
5. S. Kobayashi, O. Pinto de Carvalho, W. Schultz, Adaptation of reward sensitivity in orbitofrontal neurons. *J. Neurosci.* **30**, 534–544 (2010).
6. A. Rustichini, K. E. Conen, X. Cai, C. Padoa-Schioppa, Optimal coding and neuronal adaptation in economic decisions. *Nat. Comm.* **8**, 1208 (2017).
7. G. Paxinos, X.-F. Huang, A. Toga, The Rhesus Monkey Brain in Stereotaxic Coordinates (Academic Press, San Diego) (2000).
8. E. G. Merrill, A. Ainsworth, Glass-coated platinum-plated tungsten microelectrodes. *Med. Biol. Eng.* **10,** 662–672 (1972).
9. C. Padoa-Schioppa, J. A. Assad, Neurons in the orbitofrontal cortex encode economic value. *Nature* **441**, 223-226 (2006).
10. K. I. Tsutsui, F. Grabenhorst, S. Kobayashi, W. Schultz, A dynamic code for economic object valuation in prefrontal cortex neurons. *Nat. Comm.* **7**, 12554 (2016).

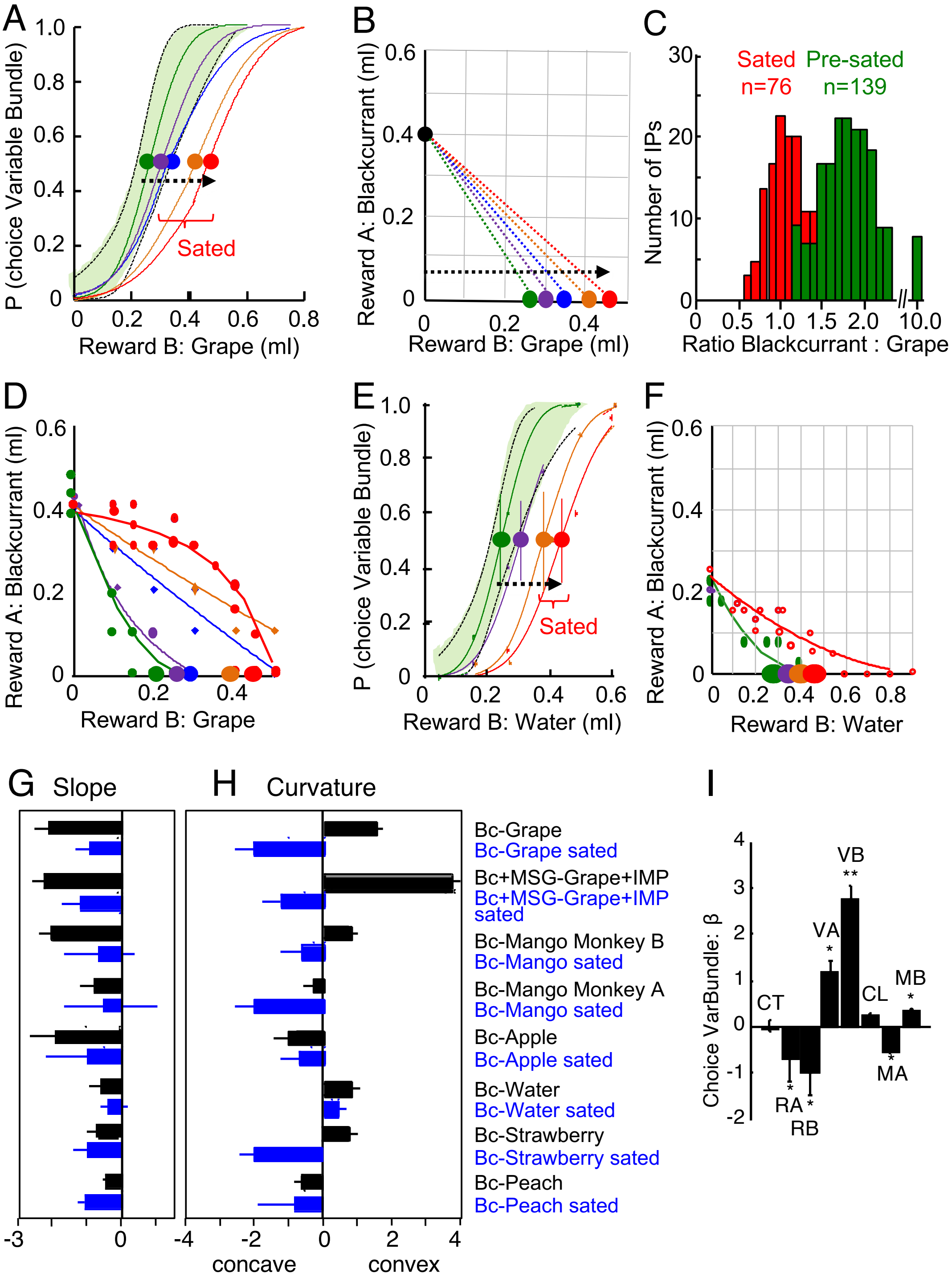

Fig. S1. Additional behavioral tests demonstrating reward-specific satiety by changes of indifference curves (IC).

(*A*) Psychophysical assessment of choice between single-reward bundles with grape juice variation (constant Reference Bundle: 0.4 ml blackcurrant juice, 0.0 ml grape juice; Variable bundle: 0.0 ml blackcurrant juice, varying grape juice). Green and violet curves inside green 95% confidence interval: initial choices; blue, orange and red curves: advancing consumption. The decrease in blackcurrant : grape juice ratio at IP was significant between the first IP and all IPs exceeding the first confidence interval (ratios of 1.9857 ± 0.0173, *n* = 139, green, vs. 1.0077 ± 0.02, orange and red; mean ± standard error of the mean, SEM; individual trial blocks: *P* = 9.6943 x 10^{7}, Kolmogorov-Smirnov test; *P* = 2.336 x 10^{-32}, Wilcoxon rank-sum test; *P* = 3.1712 x 10^{-46}, t-test;

Monkey A). Each curve and indifference point (IP) were estimated from 80 trials in a single block (Weibull fits).

(*B*) Gradually developing relative satiety for grape juice indicated by increasing choice indifference points (IP; same bundles and animal as (A)): with on-going consumption of both juices, the animal required progressively more grape juice for choice indifference against the constant Reference Bundle (from green to red). The ratio blackcurrant : grape juice quantities at IP decreased from approximately 2:1 (0.4 ml of blackcurrant juice for 0.25 ml of grape juice, black vs. green dots) to about 1:1 (0.4 ml blackcurrant for 0.45 ml grape juice, black vs. red), suggesting subjective value loss of grape juice relative to blackcurrant juice.

(*C*) Significant decrease of blackcurrant : grape juice ratio at IP with on-going consumption (same bundles as in A; Wilcoxon test). n = 139 and 76 IPs estimated in 43 trial blocks (Monkey A).

(*D*) Choice tests between two-reward Variable bundle and Reference Bundle. Gradual change with grape juice indicate slope and curvature variation of ICs between pre-satiety (green, violet) and satiety (blue, orange, red) (single session; n = 2,960 trials; 80 trials/IP; Monkey A).

(*E*), (*F*) Psychophysical tests and consumption-dependent IC change in Monkey B (constant Reference Bundle: 0.25 ml blackcurrant juice, 0.0 ml water; Variable bundle: 0.0 ml blackcurrant juice, varying water). With on-going consumption of both liquids, the animal gave up progressively more water for obtaining the same 0.25 ml of blackcurrant juice (from green to red), suggesting subjective value loss of water relative to blackcurrant juice. Same conventions as (*D*) (n = 2,400 trials; 80 trials/IP).

(*G*), (*H*) Significant IC slope and curvature changes between pre-sated and sated states with on-going consumption of individual bundles, using regular test scheme (Fig. 1*E*) on standard two-reward bundles (Bc, blackcurrant juice; MSG, monosodium glutamate; IMP, inosine monophosphate; $P = 0.0156$ and $P = 0.0313$, respectively; Wilcoxon test). The slope parameter reflected the quantity ratio blackcurrant : other liquids at IP.

(*I*) Value control by logistic regression for choice between Variable Bundle and Reference Bundle during satiety (Eq. **2**). According to significant β regression coefficients, choice of the Variable Bundle (Choice VarBundle) correlated significantly with quantity of rewards A and B in the Variable Bundle (VA, VB) and the Reference Bundle (RA, RB) and the consumed quantity of bundle rewards A (blackcurrant; MA) and B (various other liquids; MA). Choice varied insignificantly with consecutive trial number within blocks (CT) and left-right choice (CL). n = 7,243 trials pooled from several sessions; * $P < 0.05$; ** $P < 0.01$; t-test on βs.

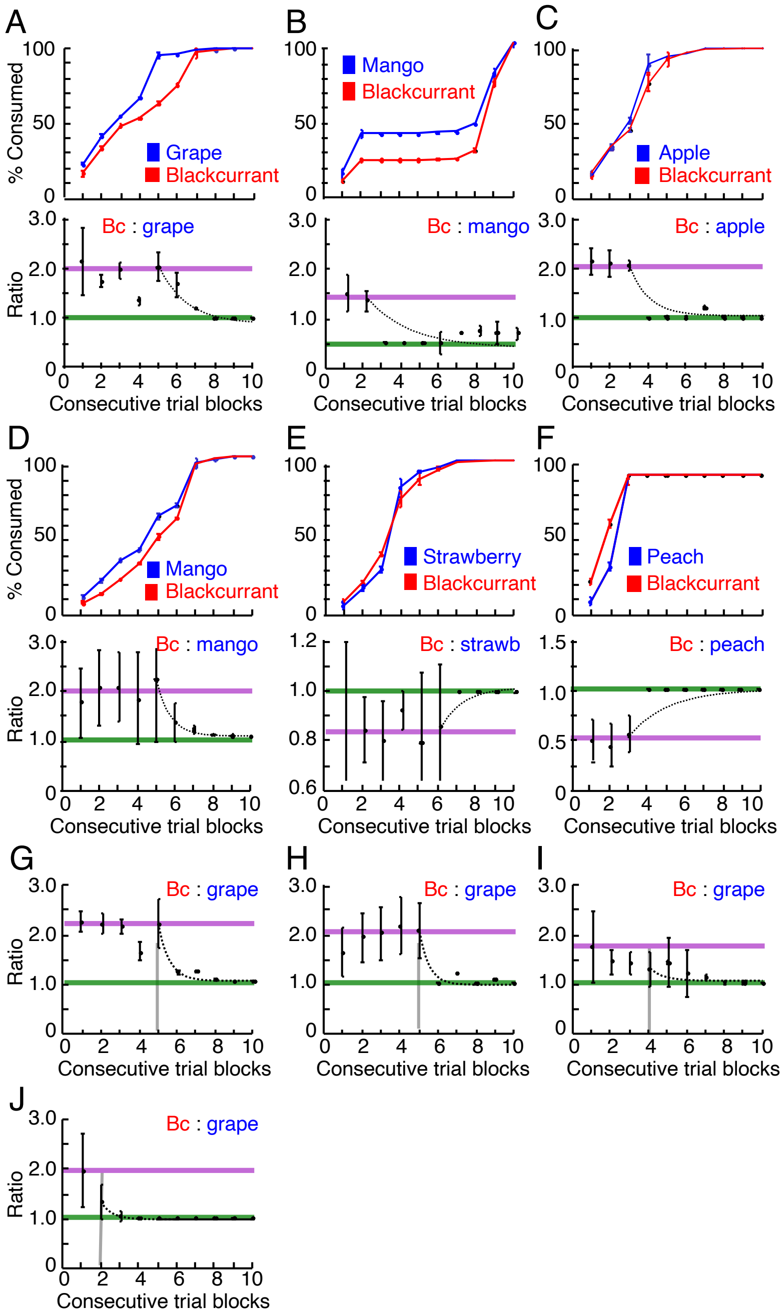

Fig. S2. Consumption profiles and ratios of further test bundles indicating reward-specific satiety.

(*A*) - (*F*) Cumulative juice consumption (top) and consumption ratios (bottom; pre-sated level: pink, sated level: green). Compared to blackcurrant juice (Bc; Reward B), on-going consumption increased significantly for the other bundle reward (Reward A) (*A*) grape juice: $P = 1.364 \times 10^{-23}$ (Monkey A; Kolmogorov-Smirnov test), (*B*) mango juice: $P = 1.3566 \times 10^{-95}$ (Monkey A); (*C*) apple juice: $P = 0.045$ (Monkey A); (*D*) mango juice: $P = 3.2564 \times 10^{-22}$ (Monkey B), changed insignificantly for (*E*) strawberry juice: $P = 0.865$ (Monkey A), and decreased significantly for (*F*) peach juice: $P = 0.035$ (Monkey B), using all trials (including bundles with two non-zero quantities). Trial numbers: (*A*): 9,812; (*B*): 5,200; (*C*): 2,160; (*D*): 12,570; (*E*): 8,201; (*F*): 440. Consumption ratio was measured only in anchor trials (each bundle contained only one liquid; see Fig. *S1A, B* for test scheme). Same conventions as Fig. 3*H, I*.

(*G*) - (*J*) Stable consumption ratios for bundle (blackcurrant juice, grape juice) during one week (from Tuesday (*G*), to Friday (*J*); Monkey A). Note more rapid ratio change on Friday (*J*).

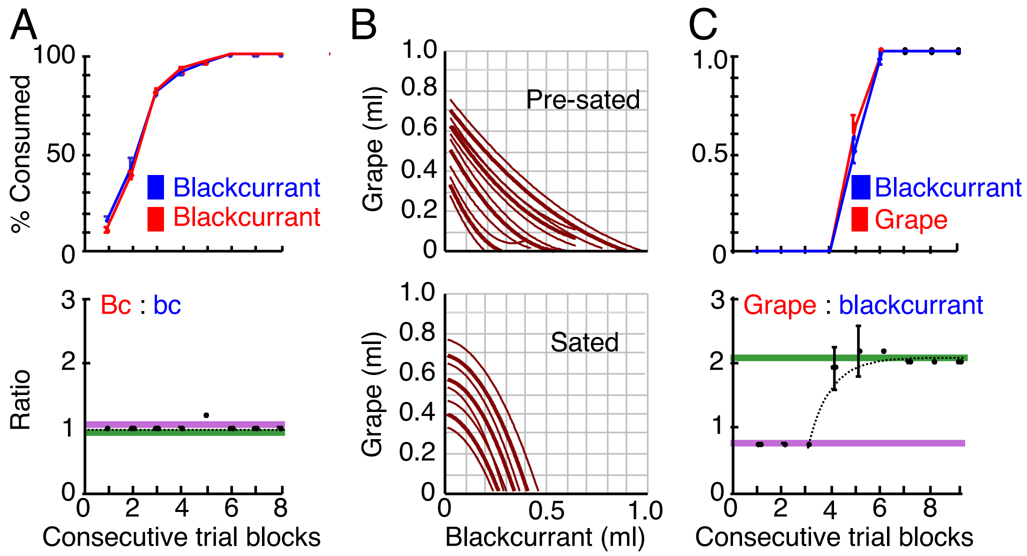

Fig. S3. Reward-specific satiety is independent of juice delivery sequence.
(*A*) Consumption profile and ratios for bundle containing blackcurrant (Bc) as both Reward A and Reward B: similar consumption and stable consumption ratio of both rewards. Pre-sated level: pink, sated level: green. For conventions, see Fig. S2.
(*B*) Indifference curves (IC) for reversed delivery sequence of grape juice (first) and blackcurrant juice (second). Bottom: consistently concave ICs suggest stronger satiety for grape juice relative to blackcurrant juice: the animal required less blackcurrant juice for giving up the same quantity of grape juice after on-going consumption of both juices, indicating lower subjective value of grape juice and suggesting grape satiety.
(*C*) Consumption profile and ratio for bundle (grape juice, blackcurrant juice) with opposite delivery sequence than usual: grape juice was delivered before blackcurrant juice.

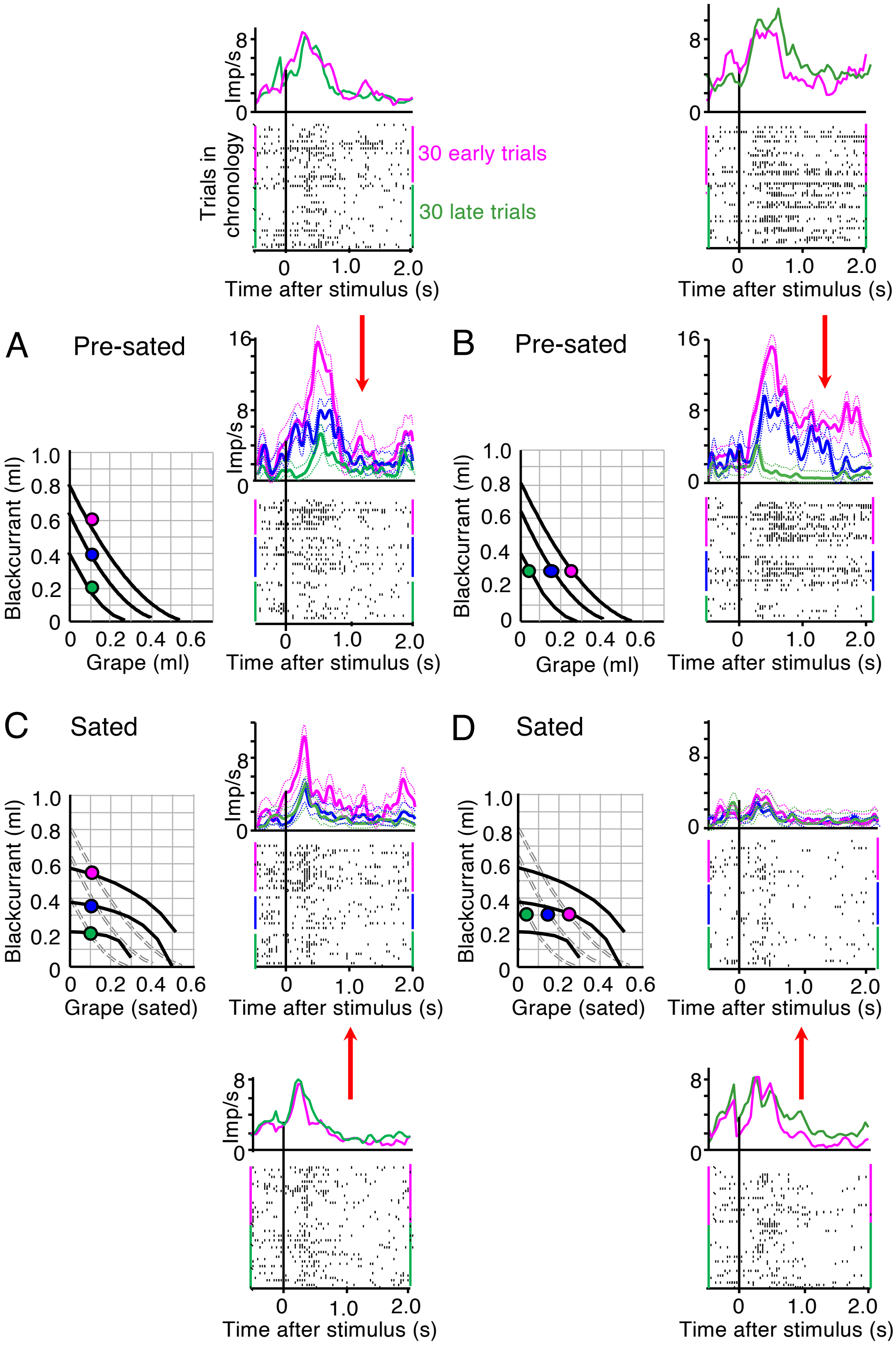

Fig. S4. Satiety-related response change in single, chosen value coding OFC neuron is unrelated to trial sequence. Separate rasters and peri-stimulus time histograms in (*A*) - (*D*) show single-neuron discharges recorded in chronological order during early and late 30 trials of a session. The combined data are shown below and above the separate displays, as indicated by red arrows, together with their 95% confidence intervals. Same data and conventions as Fig. 4.

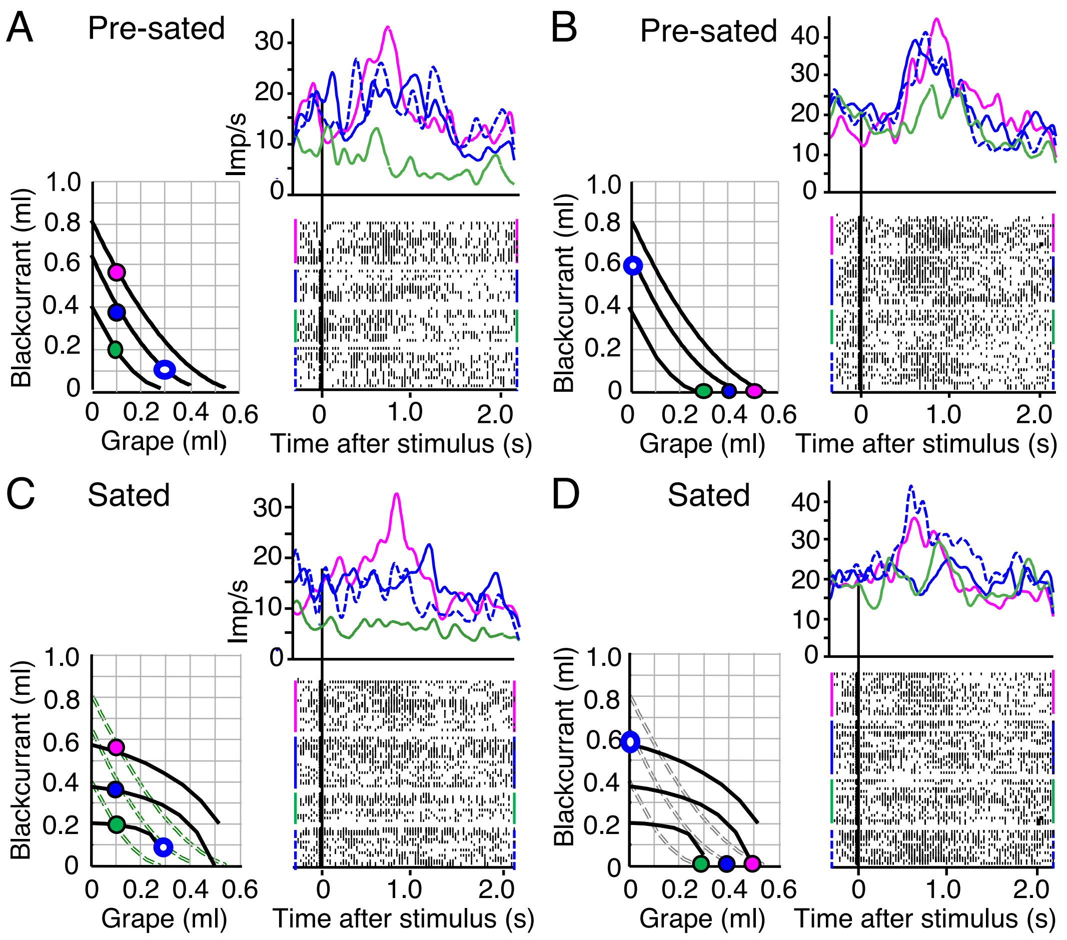

Fig. S5. Satiety-related neuronal response change during choice between two non-zero bundles.

(*A*) Significant monotonic neuronal response increase with value of chosen bundle across indifference curves (IC) before satiety (from green via solid blue to red) ($P = 0.0015$, $F(1,64) = 10.12$; $P = 6.2183 \times 10^{-13}$, $F(2,64) = 58.0$; two-way Anova: baseline vs. post-stimulus; across the 3 solid colored bundles). The animal chose between the Reference Bundle (hollow blue dot) and one of the Variable Bundles (solid colored dots). Responses to the two blue bundles on the same IC (representing equal preference) varied insignificantly despite different juice composition ($P = 0.5488$; t-test). Response to Reference Bundle (hollow blue dot) is indicated by dotted line. Binwidth 10 ms, Gaussian kernel smoothing.

(*B*) As (*A*) but for grape juice variation. Responses varied significantly across ICs with grape juice ($P = 5.0301 \times 10^{-5}$, $F(1,116) = 16.5$; $P = 1.0999 \times 10^{-9}$, $F(2,116) = 23.17$). Responses to the two blue bundles on the same IC differed insignificantly (P = 0.2622). Same color labels as (*A*).

(*C*) Despite IC change indicating satiety, the neuronal response increase across ICs remained significant ($P = 2.3892 \times 10^{-5}$, $F(1,102) = 12.921$; $P = 4.87835 \times 10^{-12}$, $F(2,102) = 48.35$). However, the two unchanged blue bundles were now on different ICs, and their responses correspondingly varied significantly ($P = 0.0028$).

(*D*) With slope and curvature change indicating satiety, the three bundles with grape juice variation were now located within only two ICs. Although the neuronal response increase across ICs remained significant ($P = 0.0071$, $F(1,106) = 4.97$; $P = 5.70 \times 10^{-7}$, $F(2,106) = 25.18$), the peak response was reduced by 25% (from 40 to 30 imp/s, red) and the three responses were closer to each other. Further, the two unchanged blue bundles were now on different ICs, and their responses now differed significantly ($P = 0.0201$). Thus, the changes of neuronal responses were consistent with the IC change indicating satiety.

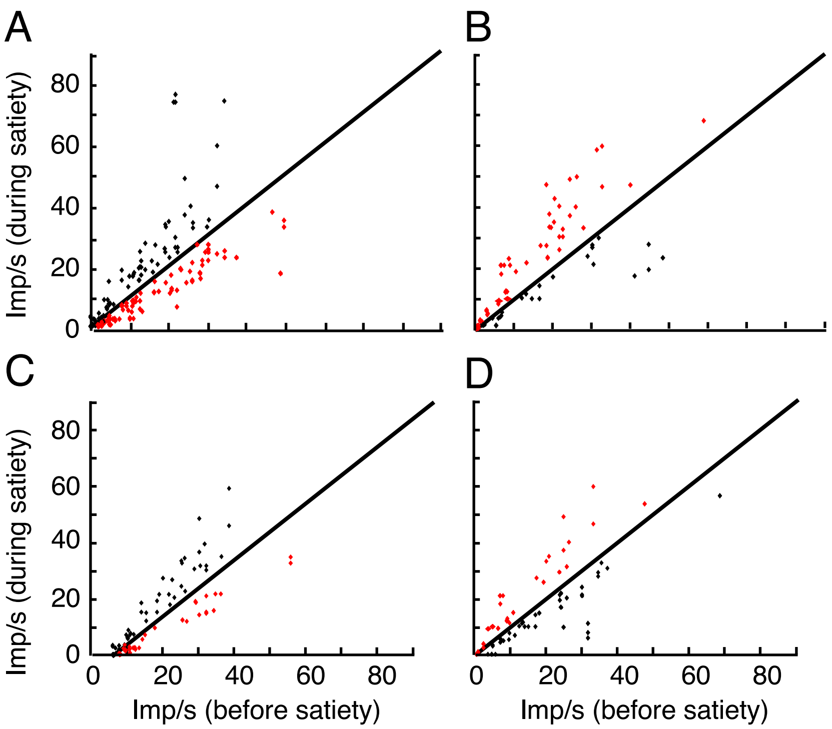

Fig. S6. Numeric display of satiety-induced neuronal response changes.
(*A*) Response changes in positive coding chosen value neurons in any of the four task epochs (Bundle stimulus, Go, Choice and Reward; Table S1) during choice over zero-reward bundle. Red: significant response decrease in population reflecting satiety-induced subjective value reduction ($P = 7.15 \times 10^{-4}$; 101 responses in 31 neurons; 1-tailed t-test). Black: significant response increase (P = 0.0014; 69 responses in 21 neurons). Imp/s: impulses/second.
(*B*) As (*A*) but for negative (inverse) value coding neurons. Red: significant response increase reflecting satiety-induced subjective value reduction (P = 0.0013; 54 responses in 15 neurons). Black: insignificant response decrease (P = 0.1274; 33 responses in 14 neurons).
(*C*) As (*A*) but for choice between two non-zero bundles. Red: response decrease (P = 0.0156; 54 responses in 16 neurons; 1-tailed t-test). Black: response increase (P = 0.0101; 57 responses in 16 neurons). Imp/s: impulses/second).
(*D*) As (*B*) but for choice between two non-zero bundles. Red: significant response increase (P = 0.0242; 31 responses in 9 neurons). Black: insignificant response decrease (P = 0.1939; 36 responses in 14 neurons).

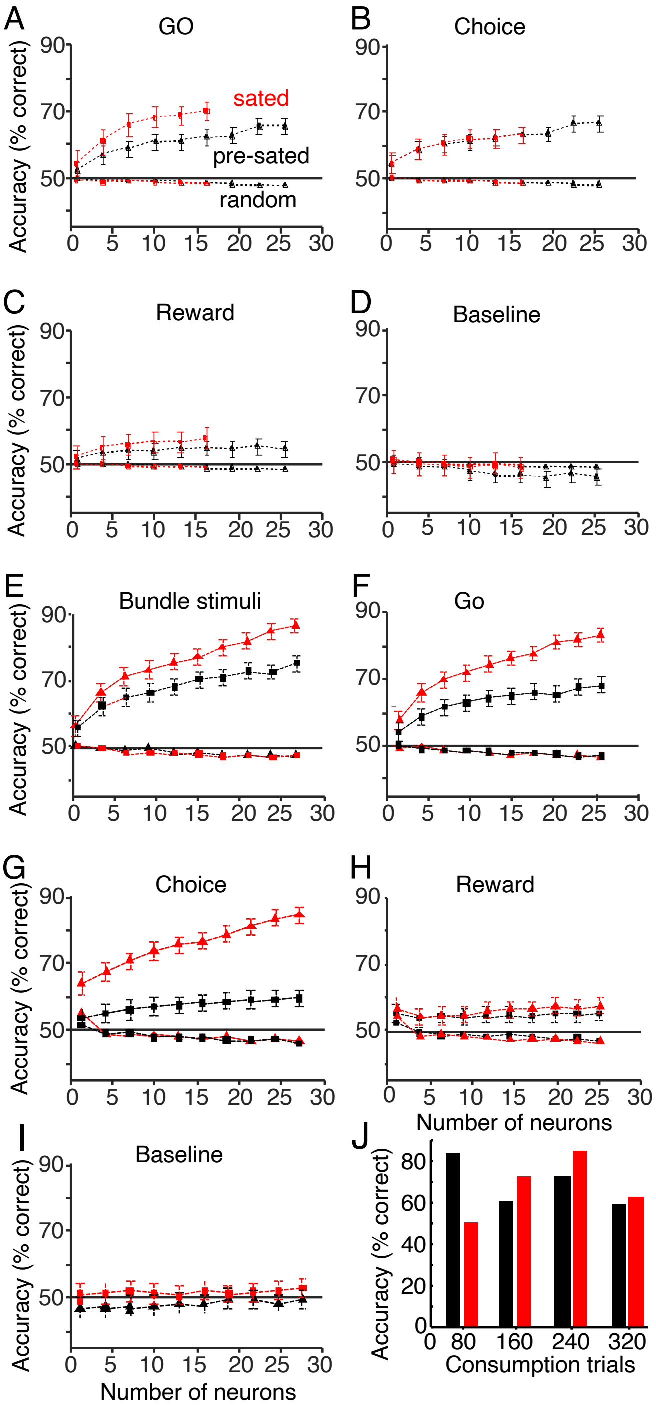

Fig. S7. Satiety-induced changes in bundle classification during specific task epochs. (*A*) - (*D*) Bundle classification by support vector machine using neuronal chosen value responses to stimuli of bundles positioned on the lowest and third lowest indifference curve, respectively (choice over zero-reward bundle). The classifier was trained on neuronal responses collected during satiety and tested during satiety (red) and before satiety (black). Baseline refers to 1 s Pretrial control epoch before Bundle stimuli. Random: control classification with shuffled assignment of neuronal responses to ICs. For conventions, see Fig. 6.
(*E*) - (*I*) As (*A*) - (*D*) but for choice prediction by neuronal responses during choice between two non-zero bundles.
(*J*) Classification accuracy of neuronal responses with advancing liquid consumption. Same data selection as for (*A*) - (*D*)) and collapsed across all task epochs. Black: before satiety, red: during satiety.

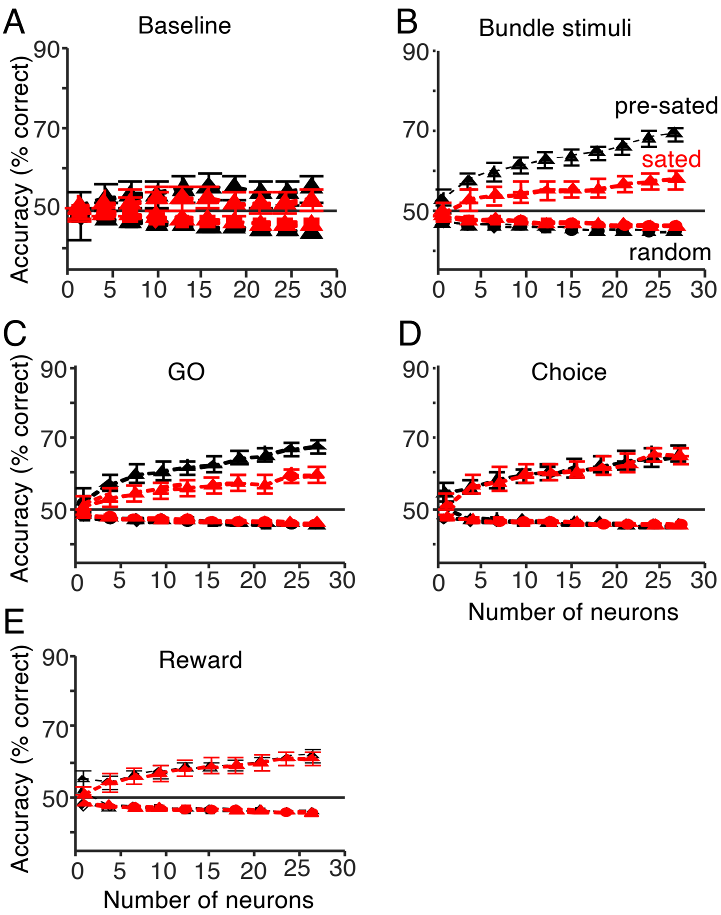

Fig. S8. Satiety-induced changes in bundle classification derived from unmodulated and unselected neuronal responses. Prediction of bundle choice between lowest and third lowest indifference curves from activity of 265 unselected neurons during the five task epochs (choice between two non-zero bundles). The classifier was trained on neuronal responses before satiety and tested for bundle distinction before satiety (black) and during satiety (red). Random: control with shuffled assignment of neuronal responses to ICs. For conventions, see Figs. 6 and S7.

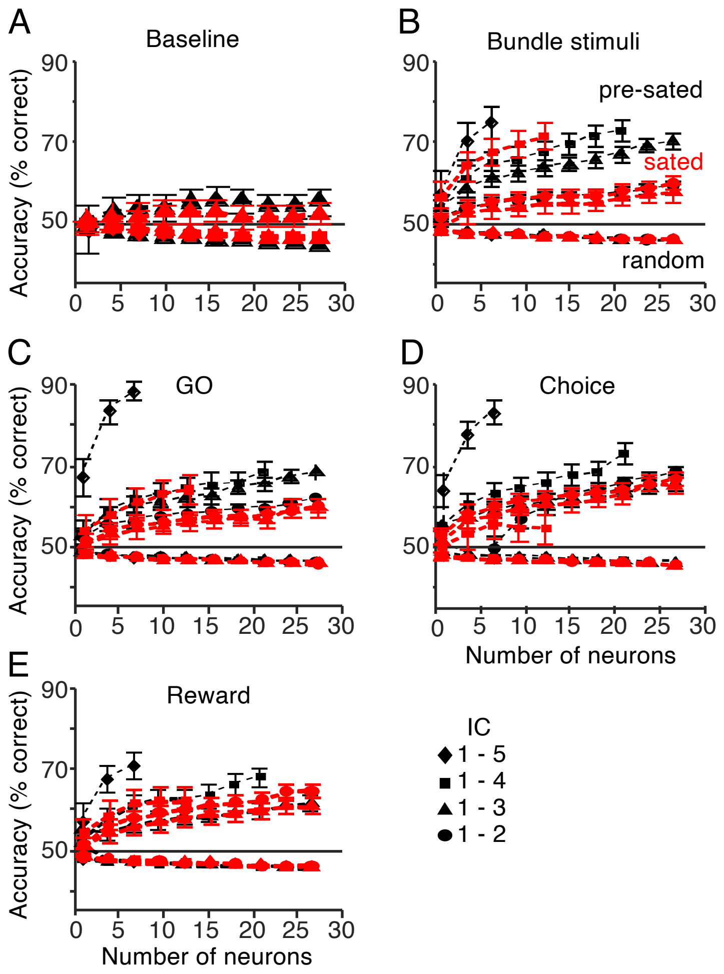

Fig. S9. Satiety-induced changes in bundle classification derived from unmodulated and unselected neuronal responses: all possible comparisons. Prediction of bundle choice from activity of 265 unselected neurons during the five task epochs (choice between two non-zero bundles). Compared to Fig. S8, all possible binary comparisons between indifference curves (IC) were tested; ICs are numbered from bottom to top, and the four comparisons are indicated by symbols. For conventions, see Fig. S8.

Table S1. Neuronal changes with on-going reward consumption during different task epochs.

Choice over zero-reward bundle						
Task epoch	Neurons tested	Neurons	Responses	Neurons	Responses	Neurons
		Response decreases		Response increases		No effects
Positive coding						
Bundle stimulus			30		21	
Go			28		17	
Choice			25		15	
Reward			18		16	
Subtotal	55	31	101	21	69	3
Negative coding						
Bundle stimulus			10		15	
Go			8		15	
Choice			8		11	
Reward			7		13	
Subtotal	31	14	33	15	54	2
Choice between two non-zero bundles						
Task epoch	Neurons tested	Neurons	Responses	Neurons	Responses	Neurons
		Response decreases		Response increases		No effects
Positive coding						
Bundle stimulus			16		16	
Go			15		15	
Choice			13		16	
Reward			10		10	
Subtotal	38	16	54	16	57	6
Negative coding						
Bundle stimulus			11		9	
Go			9		8	
Choice			8		6	
Reward			8		8	
Subtotal	32	14	36	9	31	9

The table shows data from chosen value responses, separated according to the four task epochs (Bundle stimulus, Go, Choice and Reward). Data are from all bundles tested for satiety (Reward A: blackcurrant juice, Reward B: grape juice, water or mango juice). Positive coding refers to response increase with higher subjective value before satiety, whereas negative coding refers to response

decrease with higher subjective value. Most neurons were tested both in choice over zero-reward bundle and in choice between two non-zero bundles. Changes during the Reward epoch may indiscriminately reflect changes in subjective reward value and consumption (mouth movements, sensory stimulation); no attempts were made to distinguish neuronal relationships between these factors.
